# Histamine H3 Receptor Antagonist, Thioperamide, Improves Behavioral and Neuropathological Changes Associated with Subclinical Hypersensitivity to a Cow’s Milk Allergen

**DOI:** 10.1007/s11481-025-10256-9

**Published:** 2025-12-19

**Authors:** Danielle Germundson-Hermanson, Marilyn G. Klug, Kumi Nagamoto-Combs

**Affiliations:** 1 Department of Pathology, University of North Dakota School of Medicine and Health Sciences, ND 58202-9037 Grand Forks, USA; 2 Department of Biomedical Sciences, University of North Dakota School of Medicine and Health Sciences, ND 58202-9037 Grand Forks, USA; 3 Department of Population Health, University of North Dakota School of Medicine and Health Sciences, ND 58202-9037 Grand Forks, USA

**Keywords:** Demyelination, Depression, Histamine N-methyltransferase, β-lactoglobulin, Mast cell, Cognitive dysfunction

## Abstract

Mood and behavior-related comorbidities are often reported with food allergies, an atopic condition that elevates histamine (HA) levels in tissues and circulation. However, whether allergy-induced HA directly affects the central nervous system is unclear. Previously, we demonstrated that the levels of HA and its receptor subtype, H3 receptor (H3R), were elevated in the brains of mice with subclinical cow’s milk allergy (CMA) generated by sensitizing C57BL/6J mice to a bovine whey allergen, β-lactoglobulin (BLG, Bos d 5). Furthermore, these BLG-sensitized CMA mice showed depression-like behavior associated with mast cell activation, neuroinflammation, and cortical demyelination, leading us to postulate that peripheral immune responses raised brain HA and dysregulated the neuronal histaminergic system. Hypothesizing that the autoregulatory function of H3R signaling is pivotal in eliciting altered behavior and neuropathologies, we investigated whether thioperamide, a brain-permeable H3R-selective antagonist, would attenuate the changes observed in CMA mice. Male and female CMA mice were fed a whey-containing diet for 2 weeks without or with thioperamide. While sensorimotor functions were not impaired in CMA mice of either sex, some aspects of affective and cognitive behaviors were significantly altered in males. Male CMA mice also showed more IgE-immunopositive, degranulated mast cells in the dura mater than females, regardless of thioperamide treatment. Importantly, thioperamide reduced CMA-associated behavioral and neuropathological changes in male mice, although it also uniquely affected female mice. Our results suggest that thioperamide ameliorates CMA-associated behavioral changes and neuropathologies via H3R inhibition in a sex-dependent manner.

**Graphical Abstract:**

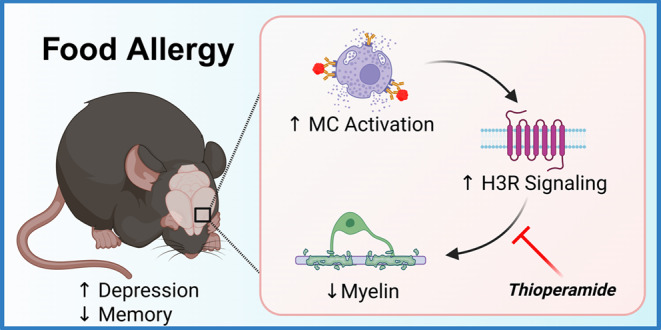

**Supplementary Information:**

The online version contains supplementary material available at 10.1007/s11481-025-10256-9.

## Introduction

Histamine (HA) is a biogenic amine known to mediate type I hypersensitivity. Upon activation of mast cells and other granulocytes, HA is rapidly released to cause edema, urticaria, and smooth muscle contraction (Akdis and Blaser 2003). HA is also produced in the central nervous system (CNS) by the neurons of the hypothalamic tuberomammillary nucleus. Additionally, HA-releasing mast cells are present in the leptomeninges and dura mater (Germundson and Nagamoto-Combs 2022; Germundson et al. 2018), although their functions are elusive.

HA release from the histaminergic (HAergic) neurons is controlled by its presynaptic autoreceptor subtype, the H3 receptor (H3R). H3R also serves as an inhibitory postsynaptic heteroreceptor for other neurotransmitter systems (Arrang et al. 1983). Other than controlling wakefulness, satiety, and thermoregulation (Lundius et al. 2010; Provensi et al. 2014), the central HAergic system influences emotion, behavior, cognition, and memory (Ito et al. 2018; Rizk et al. 2004) through its wide distribution in the brain (Lovenberg et al. 1999). Thus, altered brain levels of HA and HA receptors have been implicated in the pathogenesis of various neuropsychiatric and neurodegenerative disorders, such as narcolepsy (Kanbayashi et al. 2009), schizophrenia (Prell et al. 1995), attention deficit hyperactivity disorder (Stevenson et al. 2010), depression (Kano et al. 2004), Alzheimer’s disease (Cacabelos et al. 1989), Parkinson’s disease (Rinne et al. 2002), and multiple sclerosis (Green et al. 2017; Tuomisto et al. 1983).

HA elevation and mast cell overactivation are characteristics of mastocytosis and atopic diseases, including food allergies (Friedman et al. 1989), and these conditions are significantly correlated with anxiety (Garg and Silverberg 2014; Shanahan et al. 2014), depression (Ferro et al. 2016; Garg and Silverberg 2014; Moura et al. 2011), attention deficit disorder/attention deficit hyperactivity disorder (Ferro et al. 2016; Garg and Silverberg 2014; Shanahan et al. 2014), obsessive-compulsive disorder (Witthauer et al. 2014), and autism spectrum disorder (Lyall et al. 2015; Theoharides 2009). However, due to the lack of mechanistic evidence, whether food allergies directly contribute to the pathogenesis of these disorders remains a topic of debate.

To determine whether allergen exposure could trigger behavioral changes distinct from anaphylaxis, we previously developed a mouse model of subclinical cow’s milk allergy (CMA) and investigated the behavior of these seemingly asymptomatic “allergen-tolerant” mice. We demonstrated that acute oral challenge with bovine whey proteins produced changes in their innate behavior, accompanied by increased brain mast cell numbers and H3R expression (Germundson et al. 2018, 2020). In a follow-up study, we fed CMA mice a whey-protein-containing diet for two weeks to simulate continuous allergen consumption by subclinically hypersensitized individuals and observed apparent degranulation of brain and dural mast cells, likely in response to the allergen in the circulation (Germundson and Nagamoto-Combs 2022). HA elevation, blood-brain barrier (BBB) impairment, and cortical demyelination were also detected in the brain, suggesting that allergen consumption led to these neuropathologies through intracranial mast cell degranulation and subsequent HA elevation.

Based on the studies that supported the involvement of HA and HA receptors in the pathogenesis of multiple sclerosis (Green et al. 2017; Tuomisto et al. 1983) and oligodendrocyte differentiation (Chen et al. 2017; Rangon et al. 2018), we postulated that increased intracranial HA acting through the H3R played a pivotal role in the behavioral changes and cortical demyelination in our CMA mice. In this study, we tested whether H3R inhibition would protect against allergen-induced neurobehavioral changes in subclinically sensitized individuals during continuous allergen consumption. After sensitization to a bovine whey allergen, β-lactoglobulin (BLG; Bos d 5), male and female C57BL/6J mice were fed a whey-protein-containing diet for 2 weeks without or with thioperamide, a BBB-permeable H3R-selective antagonist. The effects of thioperamide on affective and cognitive behavior and cortical myelination were examined.

## Materials and Methods

### Animals and Treatments

Three-week-old male (*n* = 17–20 per group) and female (*n* = 10–13 per group) C57BL/6J mice were purchased from Jackson Laboratories (Bar Harbor, ME, USA, RRID: IMSR_JAX:000664). Upon arrival, mice were group-housed at 3–5 animals per cage by sex and acclimated for 1 week in a temperature-controlled specific-pathogen-free facility under a 12 h light/dark cycle. All animals were given *ad libitum* access to ultra-filtered water and a whey-protein-free rodent diet (Envigo, Indianapolis, IN, USA, Teklad 2018).

**Fig. 1:**
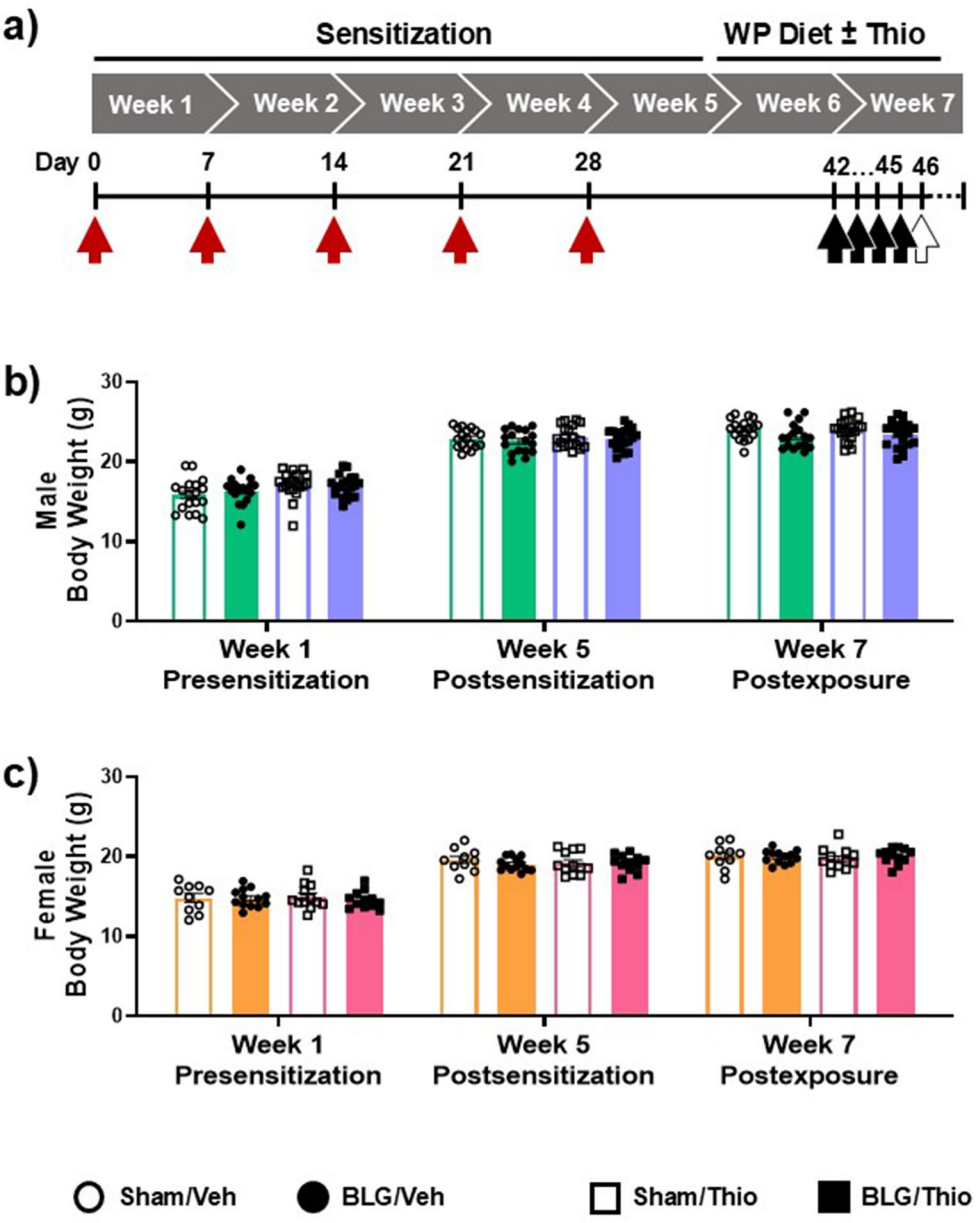
Timeline of the experimental procedure and weight gain of mice. (**a**) During the sensitization period, mice received 5 weekly intragastric gavages (red arrows) in a buffered solution containing 10 µg of cholera toxin without (sham) or with 1 mg BLG (BLG). During the subsequent allergen exposure period, all mice were placed on a diet containing whey-protein for 2 weeks with daily intragastric administration of either saline (veh; green and orange bars with open and filled circle data points) or thioperamide (Thio, 30 mg/kg; purple and pink bars with open and filled square data points). Their behavior was tested during Week 7 (black arrows) before termination (open arrow). (**b**, **c**) The body weights of sham and BLG mice were monitored throughout the experiment to assess possible food aversion. Body weights at Weeks 1, 5, and 7 are shown. (male: *n* = 17–20 per group; female: *n* = 10–13 per group)

Mice were randomly assigned to either sham or BLG sensitization groups, and sensitization was carried out as described previously to produce control and subclinical CMA mice, respectively (Germundson and Nagamoto-Combs 2022). Briefly, sham mice received 200 µL of bicarbonate buffer (pH 9.0) containing 10 µg cholera toxin as the adjuvant (List Biologicals, Campbell, CA, USA, Catalog # 100B). The sensitization was performed by intragastric gavage once a week for 5 weeks. BLG-sensitized mice (BLG mice) received 1 mg of BLG (Millipore-Sigma, Burlington, MA, USA, Catalog # L0130-5G) in the adjuvant-containing buffer. During the five-week sensitization period, all mice stayed on the whey-protein-free diet (Fig. 1a). At the beginning of Week 6, both sham and BLG mice were placed on a rodent chow with 0.3% whey-protein (Envigo, Teklad 8640) and allowed unrestricted access to the whey-protein-containing diet for 2 weeks for continuous exposure to the allergen (Brishti et al. 2022; Germundson and Nagamoto-Combs 2022). During the allergen exposure period, subgroups of sham and BLG mice were treated with either 0.9% NaCl as the vehicle control or 30 mg/kg body weight thioperamide (Sindelar et al. 2004) at a concentration of 4 mg/mL (Millipore-Sigma, Catalog # T123-100MG) daily by oral gavage to ensure each mouse received the same dosage of the vehicle or thioperamide. Allergen exposure with the whey-protein diet with the vehicle or H3R antagonist continued through the behavior testing until sacrifice. All animal use was approved by the University of North Dakota Institutional Animal Care and Use Committee (UND IACUC2109-0036).

### Behavior Testing and Analyses

After one full week of allergen exposure (i.e., starting at the beginning of Week 7 on Day 42; see Fig. 1a), all mice were subjected to a battery of behavioral tests. Testing occurred within 9 h of the vehicle or thioperamide treatment. To minimize stress, behavioral tests were performed across 4 days in the order described below, and the number of tests was also limited to 1–2 tests per day with at least 1 h of rest in their home cages in between tests.

#### Open field test

The open field test was also performed since changes in overall activity and anxiety-like behavior were previously observed in our CMA mice after acute (Germundson et al. 2022; Smith et al. 2019, 2021) and chronic (Brishti et al. 2022; Germundson and Nagamoto-Combs 2022) allergen challenge. Mice were individually placed in an opaque open-field apparatus (41.28 cm width × 41.28 cm depth × 34.92 cm height with a 20 cm × 20 cm center zone; San Diego Instruments). After a 30-s acclimation period, the activity of each mouse was video-recorded for 10 min. Changes in exploration and locomotion (time mobile, distance traveled, and the number of immobile episodes) and anxiety-like behaviors (the number of entries and time spent in the center zone of the apparatus) were analyzed using the ANY-maze software (Stoelting Co., Wood Dale, IL, USA). Because mice could increasingly become accustomed to the enclosure during the test and their behavioral pattern change could be time-dependent, we analyzed the first 4 min of the testing (Germundson and Nagamoto-Combs 2022; Smith et al. 2021). The enclosures used for the open field test were thoroughly cleaned between animal testing with a detergent-based disinfectant, Process NPD (STERIS, Mentor, OH, USA).

#### Cross maze test

Spatial working memory was assessed using the cross maze test (Brishti et al. 2022; Germundson et al. 2022; Smith et al. 2021). Briefly, mice were individually placed in one of the 4 identical arms of a cross-shaped maze with opaque walls (5 cm width × 30 cm length × 15 cm height with a 5 cm × 5 cm center zone) and allowed to explore the apparatus. The entries into the four arms of the maze were video recorded for 12 min and quantified using ANY-maze Software (Stoelting Co.). An arm entry was recorded when 95% of the animal’s total body area crossed into one of the arms, while a successful alternation was defined as a set of consecutive entries into all 4 arms before re-entering a previously visited arm. The percentage of alternation was calculated using the following formula:

$$\:\%\:alternation=\frac{number\:of\:alternations}{\left(total\:entries-3\right)}\:\times\:\:100$$

#### Novel object recognition test

 Recognition memory was tested using the novel object recognition test. Mice were placed in an open-field apparatus with two identical white cubes (3.81 cm wide) for 10 min and allowed to investigate the objects freely. After returning to their home cage for 1 h, mice were again placed in the apparatus with one of the white cubes replaced by a novel black sphere (4.44 cm diameter). The time mice spent interacting with each of the 2 objects was recorded with ANY-maze software. The analysis was limited to the first 5 min of the test as mice tended to become increasingly familiar with the objects over time (Supplementary Fig. 4-1). The discrimination index was calculated using the following formula to normalize the differences in total exploration time among the treatment groups (Sivakumaran et al. 2018):

$$\:Discrimination\:index=\frac{time\:with\:novel\:object-time\:with\:familar\:object}{total\:exploration\:time\:of\:objects}$$

#### Grip strength test

To determine whether CMA-associated cortical demyelination affected limb strength in our mouse model, we used the grip strength test to assess musculoskeletal function according to the method described by Bonetto et al. (Bonetto et al. 2015) with slight modifications. Mice were lowered onto the metal grate attached to the force gauge (San Diego Instruments, San Diego, CA, USA) and allowed to grasp the bars with all four limbs. As they were gently pulled back by the tail, mice eventually let go of the bars. The force displayed on the digital gauge applied just before mice lost their grip was recorded as grip strength. The average grip strength from 3 consecutive trials was used for the final analysis.

#### Rotor-rod test

To determine whether changes in sensorimotor function were a potential effect of CMA-associated neuropathology, we used the ROTOR-ROD™ System (San Diego Instruments) to assess balance and coordination (Jones and Roberts 1968). Mice were individually placed on a rod (3.2 cm diameter) positioned 30.5 cm above the ground. After staying on the stationary rod for 10 s, mice balanced on the rod that accelerated at 1 rpm every 8 s until they fell off. The time mice stayed on the rod was recorded as the latency to fall, and the average values from 3 consecutive trials were used for the final analysis.

#### Tail suspension test

Depression-like behavior was assessed using the tail suspension test (Bioseb, Pinellas Park, FL) as previously described (Germundson and Nagamoto-Combs 2022; Smith et al. 2019), with slight modifications. Mice were suspended by their tail approximately 10 cm above the ground for 6 min, and their movements that reflected their attempts to escape from the upside-down position were video recorded. A 3-cm piece of plastic straw was used on the tail to reduce flexibility because the C57BL/6 strain mice tend to climb their tail to regain their upright position (Mayorga and Lucki 2001). The total time immobile and latency to the first immobile episode were manually validated for each mouse by a blinded observer. Greater time spent immobile and shorter latency to the first immobile episode were considered indicators of depression-like behavior (Steru et al. 1985).

### Blood and Tissue Collection

After the completion of all behavioral testing in Week 7, mice were euthanized by CO_2_ asphyxiation. The pleural cavity was opened, and blood was collected into microfuge tubes and EDTA-coated tubes (Sarstedt, Inc., Newton, NC, USA) via cardiac puncture. After centrifugation at 2,000 × *g*, serum and plasma samples were collected and stored at − 80 °C until use.

Prior to harvesting tissue samples, mice were perfused with phosphate-buffered saline (PBS; pH 7.4) to clear the blood. The calvaria and the attached dura mater were carefully removed from the brain and the rest of the skull. The whole brain was removed from the base of the skull and bisected sagittally. The calvaria and left hemisphere were immersion-fixed in 4% paraformaldehyde (PFA, pH 7.4) for 24 h at 4 °C. The right hemisphere was microdissected into nine regions that included the olfactory bulb, frontal cortex, striatum, parietotemporal cortex, hippocampus, thalamus/hypothalamus, midbrain, cerebellum, or brainstem (Germundson and Nagamoto-Combs 2022), snap-frozen in liquid nitrogen, and stored at − 80 °C until use.

### Enzyme-linked Immunosorbent Assays (ELISAs)

#### BLG-specific and total IgE and IgG1

Allergen-specific immunoglobulins were detected according to the protocol previously described (Germundson and Nagamoto-Combs 2021) with the following modifications. The EIA/RIA Stripwells for microplates (Corning, Inc., Corning, NY, USA, Catalog # 2580) were coated with 2 µg/mL BLG in a sodium carbonate/bicarbonate buffer (pH 9.5) overnight at 4 °C. After blocking with Assay Buffer (Invitrogen Support Pack Standard, Thermo Fisher, Waltham, MA, USA; Catalog # BMS412), terminal plasma samples were diluted to 1:40 and incubated with protein G-coated plates (Thermo Fisher Scientific, Catalog # 15133) for 1 h at 37 °C to adsorb IgG. The resulting supernatant was subsequently used for BLG-specific IgE detection. For BLG-specific IgG1 detection, plasma was diluted 1:40 and used without the protein G adsorption step. Allergen-specific IgE and IgG1 were detected using a biotinylated rat anti-mouse IgE (eBioscience, San Diego, CA, USA, Cat # 13–5992-82) or IgG1 (eBioscience, Catalog # 13–4015-82) secondary antibody and avidin HRP with 3,3’,5,5’-Tetramethylbenzidine (TMB) as the substrate (Invitrogen, Thermo Fisher Scientific, Catalog # BMS412). The reaction was terminated with Stop Solution (Invitrogen Support Pack Standard, Thermo Fisher Scientific, Catalog # BMS412), and the plates were immediately read at 450 nm and 550 nm on an ELx800 Universal Microplate Reader (BioTek Instruments, Winooski, VT, USA). The total IgE and IgG levels in plasma were quantified using IgE and IgG Mouse Uncoated ELISA Kits (Invitrogen, Thermo Fisher Scientific, Catalog # 88–50460-88 and 88–50400-88). Plasma samples were diluted to 1:50 and 1:20,000, respectively, and used according to the manufacturer’s specifications.

#### Histamine (HA) and histamine-N methyltransferase (HNMT) ELISAs

Terminal plasma samples were diluted to 1:10 to measure HA and its metabolite, N-methylhistamine (NMHA), in the circulation. To measure these analytes in brain tissues, we used 5 µg of proteins from brain lysates. The only HA metabolic enzyme in the brain, HNMT, was measured from 5 µg of brain lysate proteins. The Histamine ELISA kit (Abcam, Waltham, MA, USA, Catalog # ab213975) was used to quantify both HA and NMHA, and the Mouse Histamine N-methyltransferase ELISA Kit (MyBioSource.com, San Diego, CA, USA, Catalog # MBS2888439) was used to measure HNMT levels according to the respective manufacturer’s instructions.

### Liquid Chromatography-Mass Spectrometry (LC-MS)

Snap-frozen right brain hemispheres were sent to the Vanderbilt Neurochemistry Core Laboratory (Nashville, TN, USA) for a Targeted Neurotransmitter Panel Analysis using LC-MS to measure the levels of HA alone.

### Histology

#### Tissue embedding and sectioning

PFA-fixed left-brain hemispheres were embedded in a gelatin matrix (Nagamoto-Combs et al. 2016), cryoprotected in 30% sucrose in PBS (pH 7.4), and frozen-sectioned on a Leica SM2000R sliding microtome (Leica Biosystems, Deer Park, IL, USA) at 40 μm. Tissue sections were preserved in a cryoprotectant containing 30% (w/v) sucrose and 30% (v/v) ethylene glycol in 0.1 M phosphate buffer (pH 7.4) and stored at − 20 °C until use.

#### Immunofluorescence staining of the brain

For immunostaining, tissue sections were rinsed in PBS to remove the cryoprotectant and blocked with 0.5% (w/v) BSA, 0.1% (v/v) Triton X-100, and 10% normal goat serum for 1 h at room temperature. The brain sections were subsequently incubated with a rabbit anti-mouse neurofilament heavy chain (NF-H) antibody (Novus Biologicals, Centennial, CO, USA; Catalog # NB300-135, RRID: AB_350460) at a 1:3,000 dilution overnight at 4 °C. Following thorough washing, tissue sections were incubated with an Alexa 488-conjugated donkey anti-rabbit secondary antibody (Thermo Fisher Scientific, Catalog # A21206, RRID: AB_2535792) for 2 h at room temperature. For myelin visualization, washed sections were further stained with FluoroMyelin™ (FM) Red stain (Invitrogen, Thermo Fisher Scientific, Catalog # F34652) for 1 h at room temperature. The brain sections were mounted on glass slides and coverslipped with the non-setting VECTASHIELD^®^ Antifade Mounting Medium with DAPI (Vector Laboratories, Burlingame, CA, USA).

#### Quantification of brain immunofluorescence staining

The immunoreactivity of NF-H and FM staining were quantified as previously described (Germundson and Nagamoto-Combs 2022; Germundson et al. 2018). Briefly, photomicrographs of NF-H and FM-stained brain sections from each animal (*n* = 3) were taken from the cingulate cortex across 3 serial sections using a 10× objective on a Keyence BZ-X fluorescence microscope (KEYENCE America, Itasca, IL, USA) while maintaining constant exposure settings across experimental groups. Single-channel image files of each stain were imported into Adobe Photoshop (Adobe Systems Inc., San Jose, CA, USA), converted to grayscale, and the unstained background was set to a uniform black for all images using the curves histogram. The mean gray value of the staining in the cingulate cortex was divided by the area and expressed as optical density (OD).

#### Immunofluorescence staining of the dura mater

PFA-fixed dura samples were carefully removed from the calvaria and whole-mounted on subbed glass slides. Dura samples were rehydrated in ddH_2_O and further fixed in zinc formalin fixative (Millipore-Sigma) for 1 h at room temperature. The tissues were then incubated in a Tris–EDTA buffer (pH 9.0) containing 0.01% Tween 20 overnight at 37 °C for antigen retrieval. The dural tissues were blocked as described above and incubated with monoclonal rat anti-mouse CD117 (c-kit; eBioscience, Thermo Fisher Scientific, Catalog # 14–1171-82, RRID: AB_467433) and polyclonal goat anti-mouse IgE (Novus Biologicals, Catalog # NB7531, RRID: AB_10125910) primary antibodies at a 1:200 dilution overnight at 4 °C. Alexa 647-conjugated donkey anti-rat (Jackson Immunoresearch, Catalog # 712-605-153, RRID: AB_2340694) and DyLight™ 488-conjugated horse anti-goat (Vector Laboratories, Catalog # Dl-3088) secondary antibodies were diluted 1:800 and incubated with the tissue for 2 h at room temperature. The dural samples were thoroughly rinsed and coverslipped as described above.

#### Quantification of mast cells in the dura mater

Mast cells were quantified as described previously (Germundson and Nagamoto-Combs 2022). Briefly, CD117- and IgE-immunopositive cells were visualized under a Keyence BZ-X fluorescence microscope (KEYENCE America). A blinded experimenter counted the total number of fluorescently labeled cells in each of the 20 fields examined at 40× magnification. Mast cells were identified by their immunoreactivity to CD117, size of ~ 20 μm, and granular appearance. The mean of the 20 fields per tissue section was calculated as the average mast cell number per field. The percentage of IgE-immunopositive mast cells was calculated from the number of mast cells with colocalization of CD117 and IgE labels divided by the total number of CD117 immunopositive cells as “sensitized” mast cells.

### Statistical Analyses

Each set of data from all experimental groups was analyzed initially by a 3-way ANOVA using the SAS statistical package with the General Linear Models procedure, and the F- and p-values were summarized in a table. The main effects of the independent variables (sensitization status, sex, and thioperamide treatment) and interactions among them were further analyzed with post hoc multiple comparisons to identify the significance-driving factor(s) and indicated with bold numbers if found significant. A graph including all experimental groups was plotted for each set of data, and significant differences between particular sets of groups were indicated with numeric p-values within the graph. To determine differences among sex-matched groups, we also performed a 2-way ANOVA within each sex, and statistical significance was indicated by asterisks within each graph (alone or with the p-values from 3-way ANOVA multiple comparisons). Where appropriate, additional graphs depicting significant main effects and/or interactions were plotted as Supplementary Figures and analyzed using Student’s t-tests or 2-way ANOVA with Fisher’s least significant difference (LSD) tests, respectively. The ROUT method (Q = 1%) was used to identify outliers within each treatment group, and the values from identified outliers were removed from the final analyses. The post hoc statistical analyses were performed using GraphPad Prism v10.0 software (GraphPad Software, Inc., La Jolla, CA, USA), and the associated graphs are provided as Supplementary Figures. A *p*-value of less than 0.05 (*p* < 0.05) was considered statistically significant.

## Results

### Effects of BLG Sensitization Without or With Thioperamide On the Growth and Hypersensitivity Status of Mice

**Fig. 2:**
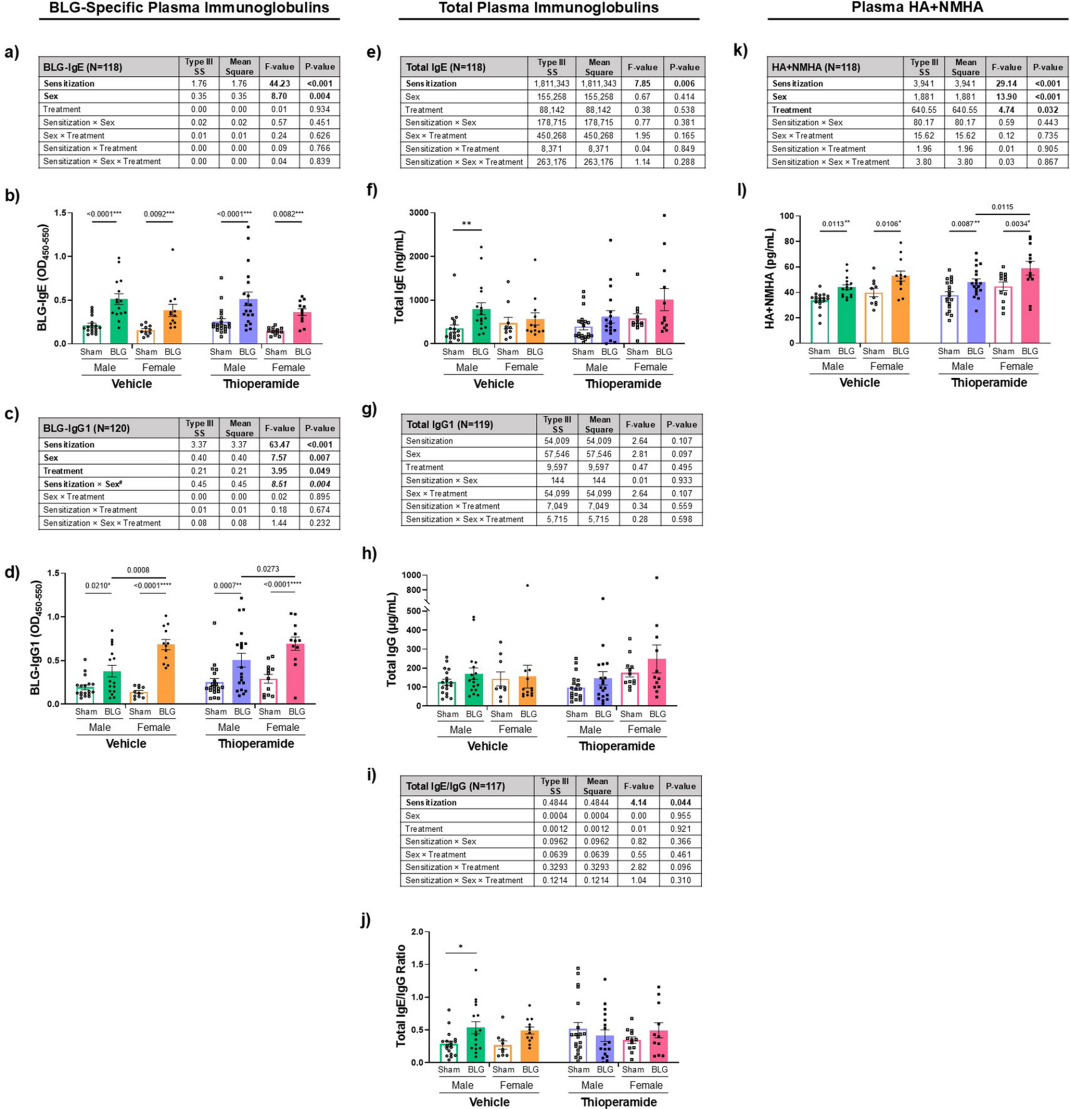
The levels of BLG-specific and total immunoglobulins and histamine (HA) and n-methylhistamine (NMHA) in the plasma. The circulating levels of BLG-IgE (**a** &** b**), BLG-IgG1 (**c** & **d**), total IgE (**e** & **f**), total IgG (**g** &** h**), total IgE/IgG ratio (**i** & **j**), and HA + NMHA (**k** &** l**) were quantified from the terminal plasma samples from male and female sham or BLG-sensitized mice treated either with the vehicle or thioperamide. The individual data values were calculated by subtracting the reference optical density (OD) values at 550 nm from the respective detection OD values at 450 nm. The IgE/IgG ratios were calculated by dividing the total IgE by the total IgG (l & j). All experimental groups were first analyzed by a 3-way ANOVA to identify significant main effects and interactions of sensitization, sex, and thioperamide treatment (treatment) as the independent variables, and the results were tabulated (a, c, e, g, i & k). The bold and italicized bold numbers indicate the F- and *p*-values of significant main effects and interactions, respectively (see Supplementary Figs. 2-1, 2-2, & 2-3 for post hoc analyses). Individual data values were also plotted to visualize the experimental outcomes (b, d, f, h, j & l). Each bar indicates the group average values ± SEM (male: *n* = 17–20 per group; female: *n* = 10–13 per group). Numbers between two bars indicate significant *p*-values from post hoc multiple comparisons. Sex-disaggregated analyses were also performed by 2-way ANOVA, and asterisks between two bars indicate significance (*p < 0.05, **p < 0.01, ***p < 0.001, ****p < 0.0001)

BLG-sensitized CMA mice did not show apparent physical symptoms indicative of food allergy-related reactions during the experimental period, as previously reported (Germundson and Nagamoto-Combs 2022). Both male and female BLG mice gained weight after sensitization (Week 5) and during allergen exposure and thioperamide (Thio) treatment (Week 7) at a similar rate to their sham counterparts (Fig. 1, b & c). Nevertheless, BLG-specific IgE (BLG-IgE) was significantly elevated in the sensitized mice overall, regardless of sex or thioperamide treatment (Fig. 2a; *p* < 0.001, 3-way ANOVA; also see Supplementary Fig. 2-1a). Multiple comparisons showed that the sensitization-induced increases in BLG-IgE were significant compared to their sex- and treatment-matched sham groups (Fig. 2b, significance indicated with numeric p-values, 3-way ANOVA). In addition to the sensitization status, sex also significantly affected BLG-IgE levels (Fig. 2a; *p* = 0.004, 3-way ANOVA), with male mice having overall greater allergen-specific antibody levels than females (Supplementary Fig. 2-1b; *p* = 0.0398, Student’s t-test). Thioperamide treatment did not affect the sensitization-induced BLG-IgE levels (Fig. 2, a & b).

Likewise, BLG-IgG1 levels were similarly affected by sensitization and sex (Fig. 2c; sensitization: *p* < 0.001; sex: *p* = 0.007, 3-way ANOVA; also see Supplementary Fig. 2-1, c & d). Furthermore, there was a significant interaction between sex and sensitization (*p* = 0.004). Indeed, BLG-IgG1 levels were significantly higher in sensitized females than sensitized males (Fig. 2d; green vs. orange filled bars, *p* = 0.0008, 3-way ANOVA). The interaction of sex and sensitization was further tested among the male and female subgroups (Supplementary Fig. 2-1e). While no difference in the mean values for BLG-IgG1 was observed between male and female sham groups (male sham = 0.22 ± 0.03 vs. female sham = 0.22 ± 0.03; *p* = 0.9761, 2-way ANOVA), the difference was significant for BLG-sensitized males and females (male BLG = 0.45 ± 0.05 vs. female BLG = 0.69 ± 0.05; *p* = 0.0002, 2-way ANOVA), even though the level was also elevated in sensitized males compared to their respective sham mice (Fig. 2d; open and filled green bars, *p* = 0.0210, 3-way ANOVA, ****p < 0.0001, 2-way ANOVA). To note, thioperamide seemed to elevate the overall levels of BLG-IgG1 slightly (Fig. 2c; *p* = 0.049, 3-way ANOVA), although the antagonist did not alter the sensitization-induced BLG-IgG1 levels within each sex (Fig. 2d). These results indicated that, while thioperamide treatment during the allergen exposure did not influence the production of BLG-specific IgE and IgG1 in both sexes, allergen-specific IgG1 might be produced more preferentially in female mice than in males with allergen exposure.

We also measured total IgE and IgG from terminal plasma samples to assess whether BLG sensitization and thioperamide treatment affected overall immunoglobulin production (Fig. 2, e-j). In a sex-aggregated analysis, a significant effect of BLG sensitization on total IgE levels was found (Fig. 2e; *p* = 0.006, 3-way ANOVA; also see Supplementary Fig. 2-2a). Within males, total IgE in vehicle-treated BLG mice was significantly greater than in sham mice (Fig. 2f; open vs. filled green bars: **p = 0.0063, 2-way ANOVA), but the difference between vehicle-treated female sham and BLG mice was not significant (Fig. 2f; open vs. filled orange bars). Similarly, no differences were observed among the thioperamide-treated groups. We did not find any notable differences among the groups for total IgG1 levels (Fig. 2, g & h). Additionally, while there appeared to be a main effect of sensitization on the overall IgE/IgG ratio (Fig. 2i; *p* = 0.044, 3-way ANOVA), post hoc tests showed that the difference between sham and BLG-sensitized animals did not reach a statistical significance (Supplementary Fig. 2-2b; *p* = 0.0738, Student’s t-test).

The levels of HA and its metabolite N-methylhistamine (NMHA) were also quantified in the plasma samples isolated at the time of euthanasia as an additional indicator of hypersensitivity response (Fig. 2, k & l). The sensitization status of the animals significantly affected the levels of HA + NMHA (Fig. 2k; *p* < 0.001, 3-way ANOVA; also see Supplementary Fig. 2-3a), increasing the plasma levels of HA + NMHA in sensitized mice of both sexes (Fig. 2l). Sex (*p* < 0.001, 3-way ANOVA) and thioperamide treatment (*p* = 0.032, 3-way ANOVA) were also independent variables that significantly affected the levels of the analytes (Fig. 2k). However, post hoc comparisons between male and female groups (Supplementary Fig. 2-3b) or between vehicle and thioperamide treatment groups (Supplementary Fig. 2-3c), indicated that the effect of sex (*p* = 0.0009), but not treatment (*p* = 0.1019), was significant, suggesting that other independent variables were confounding factors. Although a 3-way ANOVA multiple comparisons indicate that the difference in HA + NMHA levels between sensitized male and female mice was significant when compared within the thioperamide-treated group (Fig. 2l; purple vs. pink filled bars, *p* = 0.0115, 3-way ANOVA), the interaction between the sex and treatment was not significant (Fig. 2k). These results validated that H3R inhibition did not diminish peripheral HA release in sensitized mice during whey-protein-diet consumption.

### Effects of Thioperamide Treatment On CMA-associated Depression-like Behavior and Recognition Memory in Male Mice

#### Sensorimotor function

**Fig. 3:**
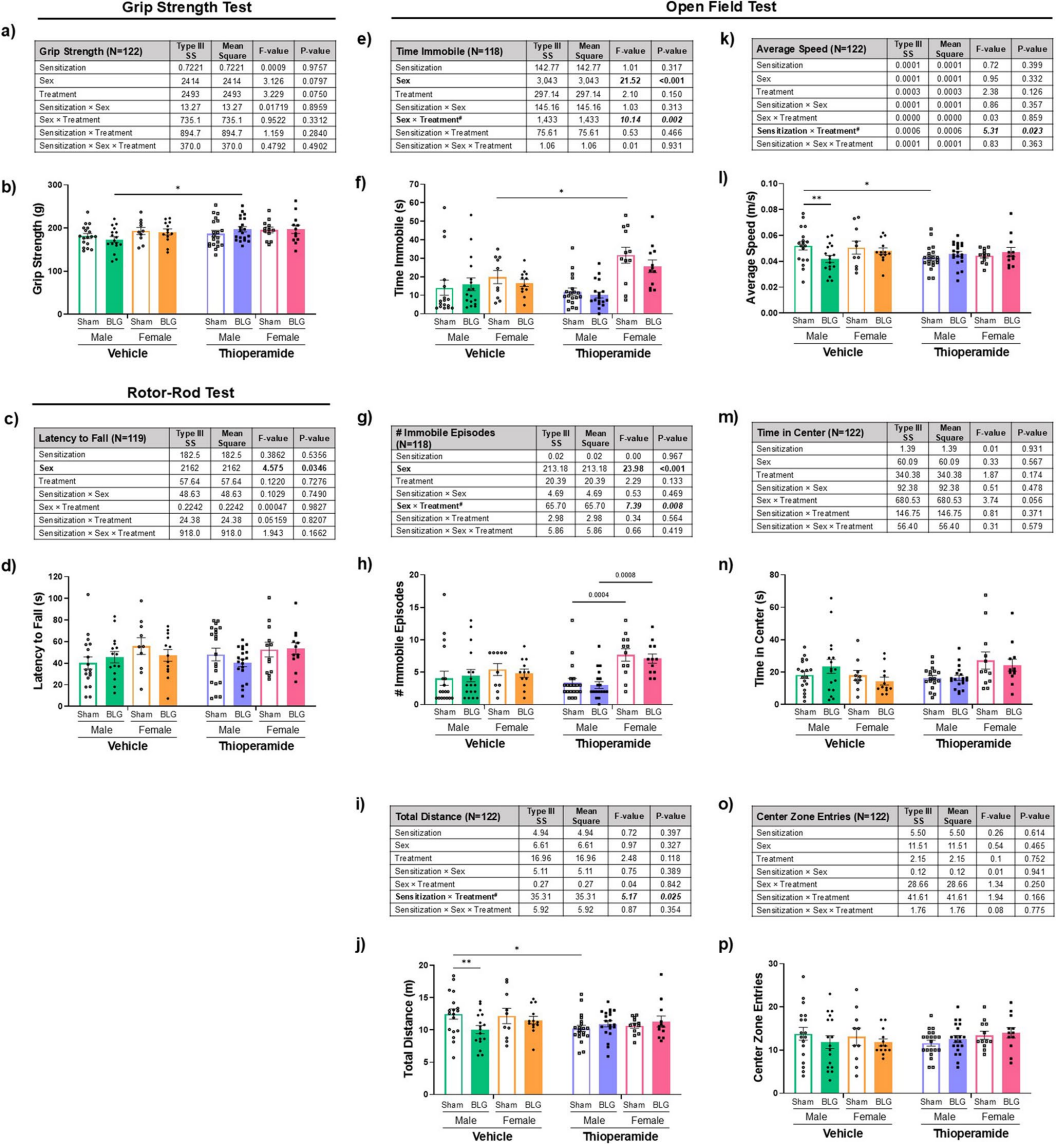
Sensorimotor function assessments with the grip strength and rotor-rod tests and locomotion and anxiety-like behavior assessments with the open field test. During Week 7, male and female sham and BLG mice treated with vehicle or thioperamide were subjected to the grip strength test (**a** & **b**) and rotor-rod test (**c** & **d**) to measure their limb strengths and sensorimotor function, respectively. Grip strength (**a** & **b**) and rotor rod tests (**c** & **d**) were conducted as 3 separate sequential trials, and the average of all trials was presented as the final result for each group. The open field test was also used to test the animals’ locomotion and anxiety-like behaviors (**e**-**p**). During the open field test, mice were allowed to move freely for 10 min to measure their general activity (**e**-**l**) and exploratory/anxiety-like behaviors (**m**-**p**). The results from the first 5 min of the open field test were presented as the final results. All experimental groups were first analyzed by a 3-way ANOVA to identify significant main effects and interactions of sensitization, sex, and thioperamide treatment (treatment) as the independent variables, and the results were tabulated (**a**, **c**, **e**, **g**, **i**, **k**, **m** & **o**). The bold numbers indicate the F- and *p*-values for sex as a significant main effect (see Supplementary Fig. 3-1, 3-2, & 3-3 for post hoc analyses). Individual data values were also plotted to visualize the experimental outcomes (**b**, **d**, **f**, **h**, **j**, **l**, **n** & **p**). Each bar indicates the group average values ± SEM (male: n = 17-20 per group; female: n = 10-13 per group). Sex-disaggregated analyses were also performed by 2-way ANOVA, and asterisks between two bars indicate significance (*p < 0.05)

 At the start of Week 7, all mice underwent a series of behavioral tests over 4 consecutive days. Mice were subjected to the grip strength test (Fig. 3, a & b) and rotor-rod test (Fig. 3, c & d) to determine whether BLG sensitization and thioperamide treatment altered motor function and coordination, respectively. No significant overall differences were detected due to sensitization (Fig. 3, a-d; 3-way ANOVA) or when BLG-sensitized mice were compared to their treatment-matched sham controls within the same sex (Fig. 3, b & d; 2-way ANOVA). However, thioperamide-treated male BLG mice showed slightly greater grip strength compared to sex-matched vehicle-treated BLG mice (Fig. 3b; *p = 0.0136, 2-way ANOVA), which was not observed in female mice (Supplementary Fig. 3-1, a & b). For the rotor-rod test, there was a small but significant main effect of sex on the latency to fall (Fig. 3c; *p* = 0.035). A post hoc analysis validated that the differences between the sexes were significant (Supplementary Fig. 3-1c, *p* = 0.0366, Student’s t-test), indicating that females generally stayed on the equipment slightly longer than males.

#### Exploratory behavior

To assess overall activities, including mobility, exploration, and location preference, the animals were also subjected to the open field test. A set of sex-aggregated analyses revealed significant effects of sex for parameters concerning immobility (Fig. 3, e & f; time immobile: *p* < 0.001; Fig. 3, g & h; number of immobile episodes: *p* < 0.001, 3-way ANOVA). Post hoc analyses comparing the sexes indicated that female mice were generally less mobile than male mice during the test (Supplementary Fig. 3-2 , a & b). Furthermore, significant interactions between sex and treatment were observed for these measures (Fig. 3 e & g; 3-way ANOVA), with thioperamide promoting immobility in sensitized females (Supplementary Fig. 3-3, a & b). In addition, significant interactions between sensitization and treatment were observed both for the total distance traveled (Fig. 3i; *p* = 0.025) and for the average speed (Fig. 3k; *p* = 0.023), with thioperamide reducing the values of these parameters in sham mice and the difference between sham and sensitized mice, regardless of sex (Supplementary Fig. 3-3, c & d).

Additional sex-disaggregated analyses confirmed that male BLG mice traveled shorter distances (Fig. 3j, open vs. filled green bars; **p = 0.0082, 2-way ANOVA) and moved at lower speeds (Fig. 3l, open vs. filled green bars; **p = 0.0073, 2-way ANOVA), but these sensitization-associated differences in distance and speed were not significant in females (Fig. 3, j & l, open vs. filled orange bars). On the other hand, the measures reflective of anxiety-like behavior with the open field test, such as time spent in the center zone (Fig. 3, m & n) and the number of center zone entries (Fig. 3, o & p), did not differ between sham and BLG mice of either sex. Interestingly, thioperamide treatment did not further reduce these activities reflective of mobility and exploratory behaviors in BLG mice.

Thus, while BLG sensitization did not impair sensorimotor function in either sex, it resulted in significant changes in some aspects of male behavior. Furthermore, the effect of thioperamide on behavior was differentially influenced by sex and the sensitization status of mice, depending on the specific behavioral parameters being assessed.

#### Depression-like behavior

**Fig. 4:**
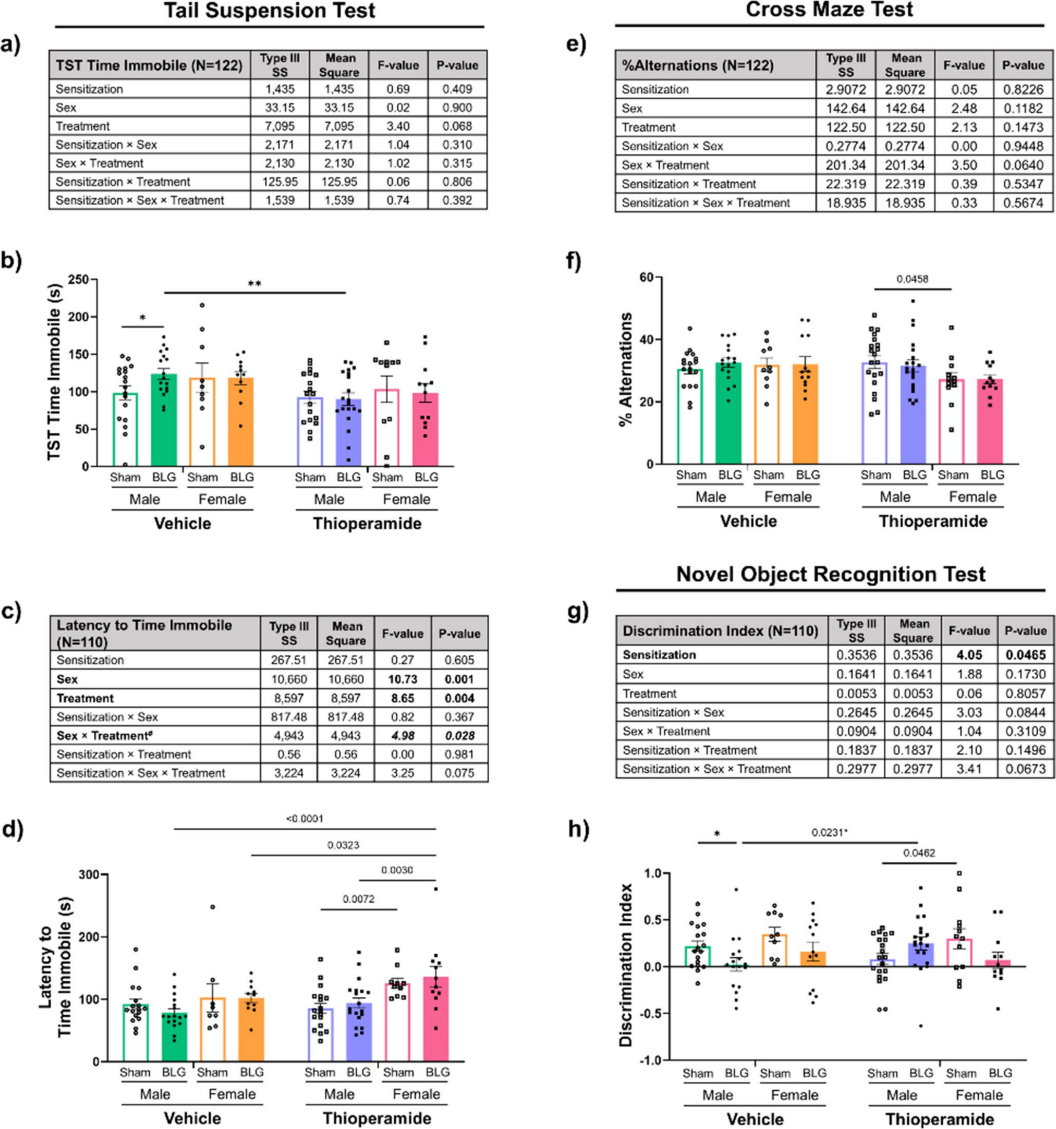
Depression-like behavior analysis with the tail suspension test and cognitive function assessments with cross maze and novel object recognition tests. During Week 7, male and female sham and BLG mice treated with vehicle or thioperamide were subjected to the tail suspension test for 6 min to assess depression-like behaviors, including the time immobile (**a **& **b**) and the latency to their first immobile episode (**c** & **d**). Additionally, mice were subjected to the cross maze test (**e** & **f**) and the novel object recognition test (**g** &** h**) to assess their short-term spatial and object-recognition memory, respectively. For the analysis of novel object recognition (g & h), the recording from the first 5 min of the 10-min testing period was used for the final results (see Supplementary Fig. 4-1 ). For each of the parameters measured, all experimental groups were first analyzed by a 3-way ANOVA to identify significant main effects and interactions of sensitization, sex, and thioperamide treatment (treatment) as the independent variables, and the results were tabulated (a, c, e, g). The bold numbers and bold italicized numbers indicate the F- and *p*-values of significant main effects and interactions, respectively (see Supplementary Figs. 4-2 and 4-3 for post hoc analyses). Individual data values were also plotted to visualize the experimental outcomes (b, d, f, h). Each bar indicates the group average values ± SEM (male: *n* = 17–20 per group; female: *n* = 10–13 per group). Numbers between two bars indicate significant *p*-values from post hoc multiple comparisons. Sex-disaggregated analyses were also performed by 2-way ANOVA, and asterisks between two bars indicate significance (*p < 0.05, **p < 0.01)

 During the behavioral testing period, mice were also evaluated for their depression-like behavior using the tail suspension test (Fig. 4, a-d). BLG sensitization did not significantly influence the overall test outcomes of the cohort, but sex and thioperamide treatment significantly affected the latency to time immobile (Fig. 4c; sex: *p* = 0.001; treatment: *p* = 0.004, 3-way ANOVA). Moreover, there was a significant interaction between sex and treatment that affected the latency (*p* = 0.028, 3-way ANOVA). A post hoc analysis of the 3-way ANOVA indicated that, among the thioperamide-treated groups, the latency to immobility was greater in female mice than in males during the tail-suspension test, regardless of their sensitization status (Supplementary Fig. 4-2e; *p* < 0.0001, 2-way ANOVA).

When sham and BLG mice were further compared within their respective sex, we observed that vehicle-treated male BLG mice spent more time immobile during the test than their sham counterparts (Fig. 4b; open vs. filled green bars, *p = 0.0384, 2-way ANOVA), as described in our prior study (Germundson and Nagamoto-Combs 2022). The male BLG group also showed a trend of becoming immobile earlier, although this measurement was not statistically different from the sham group (Fig. 4d; open vs. filled green bars, *p* = 0.2281; 2-way ANOVA). The thioperamide treatment prevented the development of depression-like behavior in male BLG mice, showing a significant reduction in their time immobile compared to their respective vehicle-treated sensitized mice (Fig. 4b; green vs. purple filled bars, **p = 0.0050, 2-way ANOVA). However, no significant differences concerning their time immobile or latency to time immobile were observed between treatment-matched female sham and BLG mice (Fig. 4, b & d). Our results showed that male and female sensitized mice displayed unique changes in behavior with the tail suspension test, and that H3R antagonism attenuated this behavioral change at least in male BLG mice.

#### Spatial and novel object recognition memory

 In our earlier study, we reported a significant decrease in myelin density in the cerebral cortex of male BLG-sensitized mice (Germundson and Nagamoto-Combs 2022). As cortical demyelination is associated with declines in memory and cognition in multiple sclerosis (Kutzelnigg et al. 2005; Lucchinetti et al. 2011), we tested short-term spatial memory and object-recognition memory using the cross maze test and novel object recognition test, respectively. When measuring the percentage of successful alternations with the cross maze as an indication of short-term spatial memory, sex was not a significant main effect (Fig. 4e; 3-way ANOVA). However, post hoc pairwise multiple comparisons within the thioperamide-treated groups indicated that female sham mice scored slightly but significantly lower than male sham mice (Fig. 4f; *p* = 0.0458, 3-way ANOVA). When males and females were further analyzed within respective sexes, however, no significant differences were observed among any of the male or female treatment groups (Fig. 4f).

With the novel object recognition test, a significant main effect of sensitization was evident on the discrimination index (Fig. 4g; *p* = 0.047, 3-way ANOVA). However, a direct comparison between overall sex-aggregated sham and BLG-sensitized groups indicated that the difference between the groups did not reach statistical significance (Supplementary Fig. 4-3, *p* = 0.1651, Student’s t-test), suggesting that the significant main effect of sensitization was influenced by the combination of sex and treatment factors. In support of this interpretation, post hoc multiple comparisons indicated that the differences in discrimination indices were significant between the vehicle-treated and thioperamide-treated male BLG groups (Fig. 4h; green vs. purple filled bars, *p* = 0.0231, 3-way ANOVA) as well as between thioperamide-treated male and female sham groups (Fig. 4h; purple vs. pink open bars, *p* = 0.0462, 3-way ANOVA). The F-value for the interactions among sensitization, sex, and treatment was 3.41, although it did not reach statistical significance (Fig. 4g; *p* = 0.0673, 3-way ANOVA),

In addition to validating the above-mentioned findings, a sex-disaggregated analysis further indicated that the discrimination index of vehicle-treated sensitized male mice was significantly lower than that of treatment-matched sham male mice (Fig. 4h; *p = 0.0379, 2-way ANOVA). The difference between the vehicle- and thioperamide-treated male BLG mice was also validated with this analysis (Fig. 4h; green vs. purple filled bars, *p = 0.0159, 2-way ANOVA), suggesting that H3R inhibition showed a beneficial effect on their short-term memory by improving object recognition. Female BLG mice showed only a decreasing trend in the discrimination index in a sex-disaggregated analysis (Fig. 4h; *p* = 0.1837, 2-way ANOVA), and thioperamide treatment did not appreciably improve this trend (*p* = 0.0924, 2-way ANOVA). Despite the tendency of sham females to perform better than their treatment-matched male counterparts (Fig. 4h), only the difference between thioperamide-treated sham males and sham females was significant (Fig. 4h; *p* = 0.0462; 3-way ANOVA).

Taken together, these findings indicated that thioperamide treatment improved some aspects of the CMA-associated behavioral changes and cognitive declines, and the effect of H3R inhibition on behavioral changes was more demonstrable in male mice.

### Effects of Thioperamide Treatment On the Activation or IgE Coupling of Intracranial Mast Cells in BLG-Sensitized Mice

Previously, we showed that mast cells, metachromatically stained with acidic toluidine blue, were prevalent throughout the dura mater of the meninges regardless of their sensitization status, with more mast cells degranulated in BLG mice (Germundson and Nagamoto-Combs 2022). We also demonstrated that numerous FcεRI-immunoreactive cells were present in both sham and BLG-sensitized animals, but the number of IgE-immunopositive cells was appreciable only in sensitized mice. To confirm that thioperamide attenuated the CMA-associated behavioral changes via its inhibitory action on H3R and not via unexpected effects on intracranial mast cell properties, we investigated whether thioperamide influenced the degranulation status of dural mast cells and their coupling with IgE. For this purpose, we co-stained dural mast cells for CD117 (c-kit) and IgE and examined their morphology to assess whether IgE-coupled mast cells had been degranulated.

#### Total mast cells in the dura

**Fig. 5:**
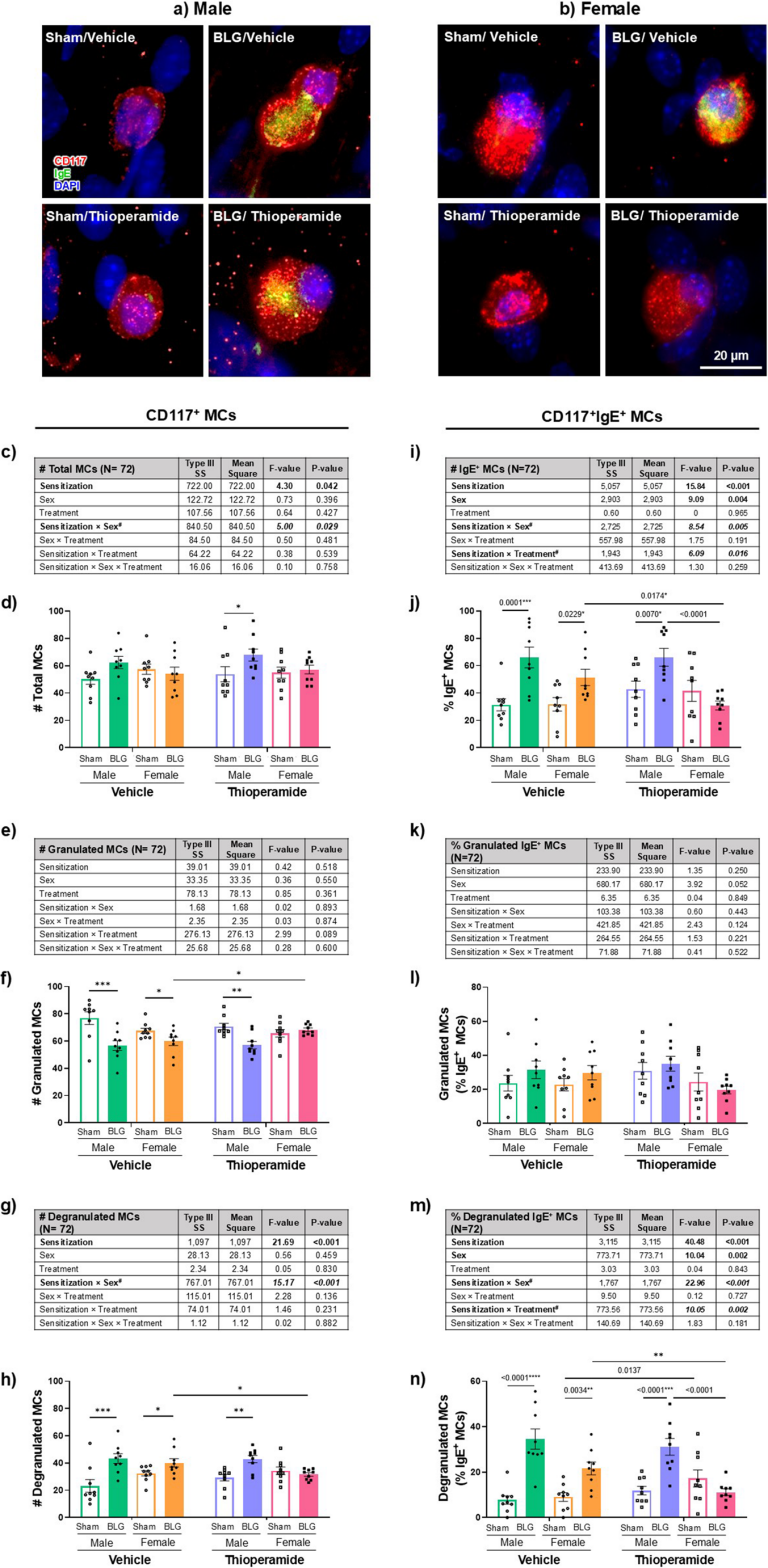
Immunofluorescence staining and quantification of IgE-coupled intracranial mast cells. Terminal dura mater tissues from male (**a**) and female (**b**) sham and BLG mice treated with vehicle or thioperamide were immunofluorescently triple-stained for CD117 (red), IgE (green), and DAPI (blue). Representative photomicrographs of mast cells (MC) are shown. The number of total CD117^+^ mast cells (**c-h**) and IgE^+^ mast cells (**i-n**) was quantified by counting the stained cells in 20 distinct microscope fields at 40× magnification, and % IgE^+^ mast cells was calculated. Scale bar = 20 μm. For each mast cell phenotype, all experimental groups were first analyzed by a 3-way ANOVA to identify significant main effects and interactions of sensitization, sex, and thioperamide treatment (treatment) as the independent variables, and the results were tabulated (c, e, g, i, k & m). The bold and italicized bold numbers indicate the F- and *p*-values of significant main effects and interactions, respectively (see Supplementary Figs. 5-1 and 5-2 for post hoc analyses). Individual data values were also plotted to visualize the experimental outcomes (b, d, f, h, j, l & n). Each bar indicates the group average values ± SEM (*n* = 9). Numbers between two bars indicate significant *p*-values from post hoc multiple comparisons. Sex-disaggregated analyses were also performed by 2-way ANOVA, and asterisks between two bars indicate significance (*p < 0.05, **p < 0.01, ***p < 0.001, ****p < 0.0001). See Supplementary Fig. 5-3 for correlation plots between the levels of BLG-IgE and the number/percentage of mast cells of distinct morphology

In our initial observation, CD117-immunopositive mast cells were found throughout the dura mater regardless of the sensitization condition or sex (Fig. 5, a & b). However, when mast cells were quantified, BLG sensitization was found to slightly but significantly influence the overall number of dural mast cells (Fig. 5c; *p* = 0.042, 3-way ANOVA). Furthermore, an interaction between sensitization and sex (*p* = 0.029) was identified, and a post hoc test revealed that the elevation in the total number of mast cells was specific to BLG males compared to sham males (Supplementary Fig. 5-1a; *p* = 0.0028, 2-way ANOVA), and the difference between male and female sensitized mice was significant (Supplementary Fig. 5-1a; male BLG = 65 ± 3 vs. female BLG = 55 ± 3; *p* = 0.0294, 2-way ANOVA), although no significant change was noted between sexes of sham mice (male sham = 52 ± 3 vs. female sham = 56 ± 3; *p* = 0.3233, 2-way ANOVA). When compared among groups within each sex, only the difference between thioperamide-treated sham and BLG male mice was significant, with 25% more mast cells in the latter group (Fig. 5d; *p = 0.0380, 2-way ANOVA).

Based on these findings, we further quantified granulated or degranulated mast cells based on their morphology. No significant effects were found on the number of granulated mast cells in a sex-aggregated analysis (Fig. 5e). When sexes were compared independently, BLG sensitization significantly decreased the number of granulated mast cells across all groups, except the thioperamide-treated female group having a higher number of granulated mast cells than their sex-matched vehicle controls (Fig. 5f; orange vs. pink filled bars, *p = 0.0166, 2-way ANOVA).

In contrast to granulated mast cells, the number of degranulated mast cells in the dura was strongly affected by sensitization status (Fig. 5g; *p* < 0.001, 3-way ANOVA) as we observed for the total mast cell numbers (see Fig. 5c). An interaction between sex and sensitization status also significantly influenced the number of degranulated mast cells (Fig. 5g; *p* < 0.001, 3-way ANOVA). Post hoc multiple comparisons indicated that BLG sensitization markedly increased the number of mast cells with the activated morphology in male sensitized mice compared to sex-matched sham and to female BLG mice (Supplementary Fig. 5-1b; male sham = 14 ± 2 vs. male BLG = 28 ± 2, *p* < 0.0001; male BLG = 28 ± 2 vs. female BLG = 20 ± 2, *p* = 0.0085, 2-way ANOVA). The difference between male and female sham groups was not significant (male sham = 14 ± 2 vs. female sham = 19 ± 1; *p* = 0.1255, 2-way ANOVA).

In a sex-disaggregated analysis, the sensitization-associated increases in the number of degranulated mast cells were apparent in the vehicle-treated mice of both sexes (Fig. 5h; male: sham vs. BLG, ***p = 0.0002; female: sham vs. BLG, *p = 0.0290, 2-way ANOVA). Although the 3-way ANOVA did not detect significant main effect or interactions of treatment, direct comparisons of vehicle-treated and thioperamide-treated groups within the same sex indicated that the H3R antagonist affected degranulated mast cell number in female mice, decreasing the sensitization-associated changes (Fig. 5h; orange vs. pink filled bars, **p = 0.0084, 2-way ANOVA), having an opposite effect as it did for granulated cells (see Fig. 5f). Thioperamide-treated sensitized male mice still showed significant increases in degranulated mast cell number compared to the treatment-matched sham mice (Fig. 5h; open and filled purple bars, *p = 0.0166, 2-way ANOVA).

#### IgE-immunoreactive mast cells in the dura

When dural mast cells were further characterized by their double-immunoreactivity to both CD117 and IgE, the overall percentage of IgE-positive (IgE^+^) mast cells was impacted not only by sensitization (Fig. 5i; *p *< 0.001, 3-way ANOVA) but also by sex (*p* = 0.004, 3-way ANOVA), with BLG sensitization and male sex independently increasing the overall percentage of IgE^+^ mast cells (Supplementary Fig. 5-2, a & b, respectively). In addition, significant interactions between sensitization and sex (Fig. 5i; *p* = 0.005) and sensitization and treatment (*p* = 0.016) were identified. When the interaction between sex and sensitization was further analyzed, the percentage of total IgE^+^ mast cells in BLG-sensitized males was significantly greater than the female BLG group (Supplementary Fig. 5-2c; male BLG = 66 ± 5% vs. female BLG = 41 ± 4%, *p* = 0.0008, 2-way ANOVA), indicating a strong sex-specific effect in terms of the sensitization and treatment interaction. In addition, post hoc multiple comparisons indicated that the percentage of IgE-coupled mast cells was elevated in vehicle-treated sensitized mice of both sexes (Fig. 5j; male: open vs. closed green bars, *p* = 0.0001; female: open vs. filled orange bars, *p* = 0.0229, 3-way ANOVA). While thioperamide did not decrease the sensitization-associated increase in the IgE^+^ mast cells in males (Fig. 5j; open vs. filled purple bars, *p* = 0.0070, 3-way ANOVA), the H3R antagonist decreased this population of mast cells in female BLG mice compared to the treatment-matched sham (Fig. 5j, open vs. filled pink bars) or vehicle-treated female counterparts (Fig. 5j, orange vs. pink filled bars, *p* = 0.0174, 3-way ANOVA), suggesting that the H3R antagonist reduces the number of allergen-responsive mast cells in female dura.

An additional morphological assessment of double immunopositive cells showed that there was no change in the percentage of IgE-coupled granulated mast cells (Fig. 5, k & l). However, large percentages of IgE-positive mast cells were degranulated in BLG-sensitized mice (Fig. 5, m & n). Similarly to our findings with the overall IgE-coupled mast cells, IgE-associated degranulated mast cells were affected independently by sensitization (Fig. 5m; *p* < 0.001, 3-way ANOVA) and sex (*p* = 0.002, 3-way ANOVA). Post hoc analyses of these factors indicated that BLG sensitization and male sex significantly increased the percentage of IgE^+^ mast cells (Supplementary Fig. 5-2, e & f, respectively). We also found strong interactions between sensitization and sex (*p* < 0.001, 3-way ANOVA), as well as sensitization and thioperamide treatment (*p* = 0.002, 3-way ANOVA). Post hoc tests validated that the BLG-sensitized male group showed an elevated percentage of degranulated IgE^+^ mast cells compared to BLG-sensitized females (Supplementary Fig. 5-2g; male BLG = 32 ± 3% vs. female BLG = 16 ± 2%, *p* < 0.0001, 2-way ANOVA). The percentage between male and female sham groups was comparable (male sham = 10 ± 1% vs. female sham = 13 ± 2%, *p *=0.6998, 2-way ANOVA). Additional analyses of the interaction between sensitization and treatment indicated that sensitization-induced increases in IgE^+^ mast cell percentage were only significant in the vehicle-treated mice and not in the thioperamide-treated groups (Supplementary Fig. 5d-h; *p* = 0.0001, 2-way ANOVA). Multiple comparisons revealed that the effect of thioperamide to reduce the sensitization-induced increases in the percent of degranulated IgE^+^ mast cells was largely observed in female BLG groups and not in male mice (Fig. 5n; green vs. purple filled bars, *p* < 0.0001, 3-way ANOVA), suggesting that sex contributes as a confounding factor to some extent.

While the sex-aggregated analysis indicated that the H3R antagonist slightly increased the percent of degranulated IgE^+^ mast cells in sham females compared to the vehicle-treated counterpart (Fig. 5n; orange vs. pink open bars, *p* = 0.0137, 3-way ANOVA), the overall the difference between the two groups was no longer significant when analyzed by 2-way ANOVA within female groups. Nevertheless, thioperamide treatment significantly reduced the percentage of degranulated IgE^+^ mast cells in BLG-sensitized females compared to the vehicle-treated female BLG mice (Fig. 5n, orange and pink filled bars, **p = 0.0084, 2-way ANOVA). These findings indicate that IgE-coupled mast cells increase in the dura mater of sensitized mice regardless of sex, but their number may be uniquely regulated, with thioperamide having a notable influence in females.

Sex differences in the effect of sensitization became apparent when the relationships of the serum levels of BLG-specific IgE with the numbers of total mast cells, degranulated mast cells, and IgE-coupled dural mast cells were examined. We found significant correlations between BLG-IgE and these mast cell measures in males (Supplementary Fig. 5-3, a-f), but such correlations were not found in female mice (Supplementary Fig. 5-3, g-l). These observations indicated that the increased number and activation of mast cells were more likely associated with BLG sensitization in males than in females. Importantly, thioperamide did not alter sensitization-induced changes in CD117-positive mast cells in males, suggesting that the attenuation of behavioral changes observed with thioperamide treatment was not due to its influence on the activation or IgE coupling of dural mast cells.

### Region-Specific Effects Of BLG Sensitization and Thioperamide on HA, N-methylhistamine, and Histamine N-methyltransferase Levels in the Brain

Because BLG sensitization significantly elevated the number of degranulated dural mast cells (Fig. 5, h & n), we predicted that excessive HA had been released into the intracranial space. Thus, we next quantified the HA levels in different brain regions. Using protein lysates from the frontal cortex (fCTX), hippocampus (HPC), and thalamus (THAL), we performed a competitive ELISA that detected both HA and its metabolite, N-methylhistamine (NMHA).

**Fig. 6:**
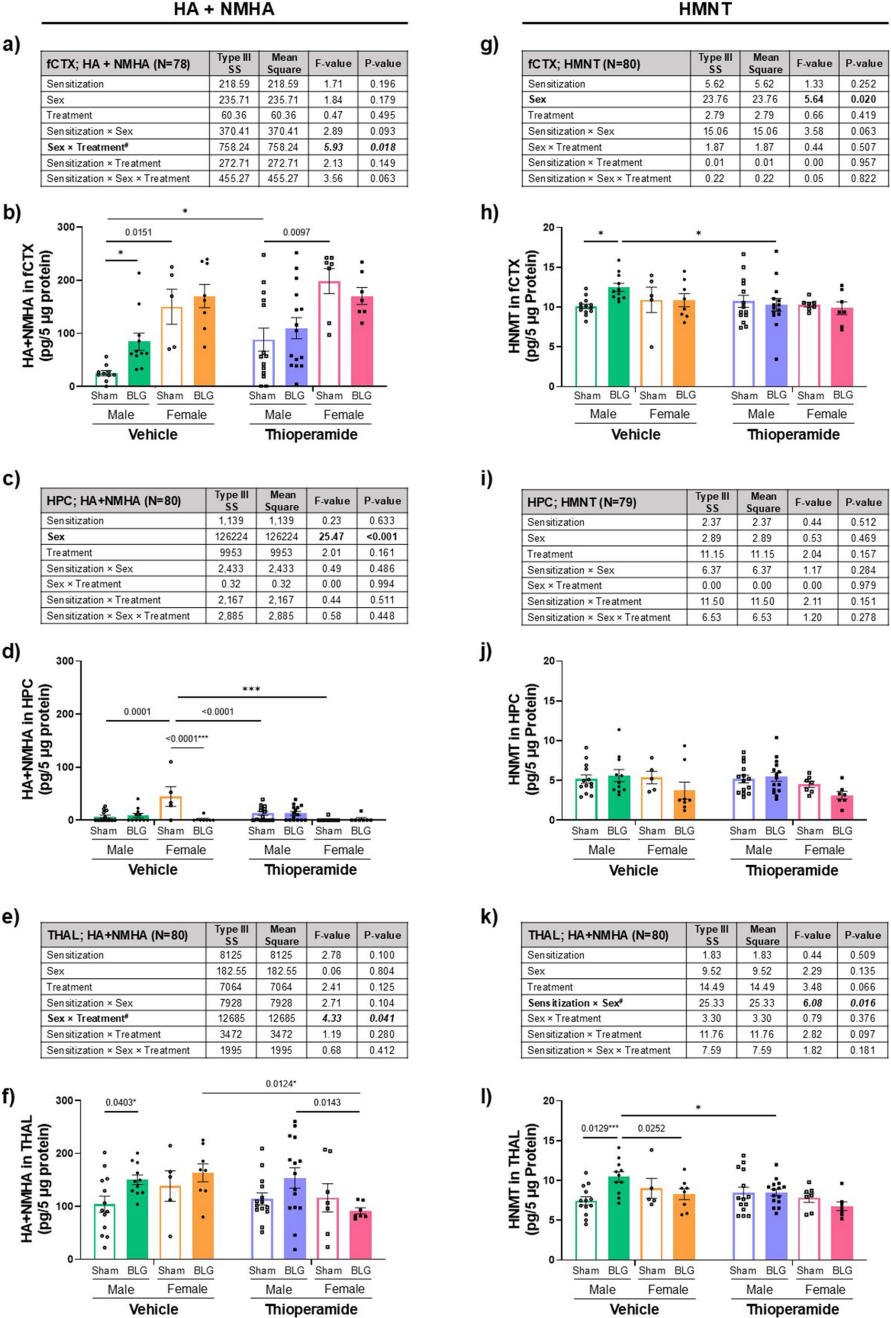
The levels of histamine (HA) and N-methylhistamine (NMHA), and histamine N-methyltransferase (HNMT) in select regions of the brain. The levels of HA and its metabolite, NMHA (**a-f**), and HA’s metabolic enzyme, HNMT (**g-l**) were quantified by ELISA using the tissue lysates from the frontal cortex (fCTX: a & b; g & h), hippocampus (HPC: c & d; i & j), and thalamus (THAL: e & f; k & l) of male and female sham and BLG mice with vehicle or with thioperamide treatment. For each brain region, all experimental groups were first analyzed by a 3-way ANOVA to identify significant main effects and interactions of sensitization, sex, and thioperamide treatment (treatment) as the independent variables, and the results were tabulated (a, c, e, g, i & k). The bold and italicized bold numbers indicate the F- and *p*-values of significant main effects and interactions, respectively (see Supplementary Fig. 6-1 for post hoc analyses). Individual data values were also plotted to visualize the experimental outcomes (b, d, f, h, j & l). Each bar indicates the group average values ± SEM (male: *n* = 12–15; female: *n* = 5–8). Numbers between two bars indicate significant *p*-values from post hoc multiple comparisons. Sex-disaggregated analyses were also performed by 2-way ANOVA, and asterisks between two bars indicate significance (*p < 0.05, ***p < 0.001). For the quantification of HA only with liquid chromatography-mass spectrometry (LC-MS), see Supplementary Fig. 6-2

When HA + NMHA levels in the three brain regions were examined by 3-way ANOVA in sex-aggregated analyses, no overall significant main effect of sex was found in the frontal cortex with 3-way ANOVA (Fig. 6a). However, there was a significant interaction between sex and thioperamide treatment in the frontal cortex (*p* = 0.018, 3-way ANOVA). Multiple comparisons showed that the levels of HA + NMHA were markedly greater in sham females than sham males, regardless of thioperamide treatment (Fig. 6b; green vs. orange open bars, *p* = 0.0151; purple vs. pink open bars, *p* = 0.0097; 3-way ANOVA). Post hoc analyses on the interaction between sex and treatment indicated that the overall amounts of HA + NMHA in the frontal cortex were greater in female groups than in male groups (Supplementary Fig. 6-1a; male thio vs. female thio, *p* = 0.0001, 2-way ANOVA). We also found that, in males, the lower analyte levels in mice were significantly elevated after thioperamide treatment (Supplementary Fig. 6-1a; male vehicle vs. thioperamide, *p* = 0.0235, 2-way ANOVA). In a sex-disaggregated analysis, we found that the analyte levels were significantly elevated in BLG male mice compared to their sham counterpart (Fig. 6b, open vs. filled green bars, *p = 0.0469, 2-way ANOVA), and thioperamide treatment increased HA + NMHA levels in the frontal cortex of sham males (Fig. 6b, green vs. purple open bars, *p = 0.0263, 2-way ANOVA).

In the hippocampus, we detected overall lower levels of HA + NMHA compared to the frontal cortex or thalamus, and sex was a main effect that significantly impacted the analyte levels (Fig. 6c; *p* < 0.001, 3-way ANOVA). However, a direct comparison between the sexes, irrespective of sensitization and treatment, showed that the difference in the HA + NMHA levels was not significant (Supplementary Fig. 6-1b). Multiple comparisons indicated that vehicle-treated female sham mice had relatively greater levels of the analyte than their male counterpart (Fig. 6d; green vs. orange open bars, *p* = 0.0001, 3-way ANOVA).

In the thalamus, as in the frontal cortex, a significant interaction between sex and treatment was also found (Fig. 6e; *p* = 0.041, 3-way ANOVA). A post hoc analysis indicated that, in vehicle-treated groups, BLG sensitization significantly induced the analyte levels in males (Fig. 6f; open vs. filled green bars, *p* = 0.0403, 3-way ANOVA), but the analyte levels were not appreciably elevated in females. Furthermore, thioperamide significantly reduced HA + NMHA levels in females but not in males (Supplementary Fig. 6-1c). Multiple comparisons specified that thioperamide treatment lowered HA + NMHA levels in female BLG mice compared to vehicle-treated female BLG mice (Fig. 6f; orange vs. pink filled bars, *p* = 0.0124, 3-way ANOVA) and to thioperamide-treated male BLG mice (Fig. 6f; purple vs. pink filled bars, *p* = 0.0143, 3-way ANOVA). A 2-way ANOVA within each sex supported that the difference between male sham and BLG mice was significant (Fig. 6f; open vs. filled green bars, *p = 0.0439, 2-way ANOVA). Unlike in the frontal cortex, however, thioperamide treatment did not elevate HA + NMHA levels in the thalamus of male sham groups, but instead, decreased the analyte levels in female sensitized groups, suggesting the H3R antagonist affected the analyte levels differentially among male and female groups in a region-specific manner. While these sets of data again highlighted additional sex-biased effects of sensitization and thioperamide treatment, they also uncovered regional variations in the levels and regulation of HA + NMHA in the brain. Nonetheless, at least in the frontal cortex and thalamus, BLG sensitization was more likely to elevate the level of HA and its immediate metabolite in male mice.

To estimate the relative amounts of HA from the ELISA results, we also measured brain HA without its metabolite, NMHA, using LC-MS (Supplementary Fig. 6-2). In contrast to the ELISA results, we detected decreased levels of HA in BLG-sensitized male CMA mice. This result supported the notion that regionally elevated HA in the brains of CMA mice had been metabolized to NMHA and detected along with the remaining HA by the HA + NMHA ELISA (Fig. 6, a-f). In addition, although HA + NMHA levels in females were comparable to males when detected by ELISA, LC-MS detected much lower levels of HA in all female groups (Supplementary Fig. 6-2), highlighting the complexity of the mechanism regulating HA levels in the brain.

To further exclude the possibility that sensitization-induced elevation of the biogenic amine in some regions of the male brain was due to decreased degradation of HA, we also measured the levels of histamine N-methyltransferase (HNMT), the only metabolic enzyme of HA in the brain (Yoshikawa et al. 2019). Although the levels of HNMT within each region were relatively similar across the experimental groups, we found that sex was a significant main effect in the frontal cortex (Fig. 6g; *p* = 0.02, 3-way ANOVA), and interaction between sensitization and sex influenced the levels of HNMT in the thalamus (Fig. 6k; *p* = 0.016, 3-way ANOVA). Indeed, further analyses indicated that HNMT levels showed slight but significant increases in the thalamus (Supplementary Fig. 6-1d; male sham = 7.9 ± 0.4 vs. male BLG = 9.3 ± 0.4, *p* = 0.0226), whereas no significant difference in the enzyme levels was found between female sham and BLG groups (female sham = 8.2 ± 0.6 vs. female BLG = 7.6 ± 0.4, *p* = 0.6374, 2-way ANOVA). Additional post hoc analyses indicated that thalamic HNMT levels between males and female BLG groups were significantly different (Supplementary Fig. 6-1d; *p* = 0.0136, 2-way ANOVA), particularly in vehicle-treated groups (Fig. 6l; *p* = 0.0252, 3-way ANOVA). No significant main effects or interactions were identified in the hippocampus.

When sexes were compared independently, we found that HNMT was increased by BLG sensitization in both the frontal cortex (Fig. 6h; *p = 0.0167, 2-way ANOVA) and thalamus (Fig. 6l; ***p = 0.0010, 2-way ANOVA) of vehicle-treated male mice, while thioperamide reduced only the sensitization-induced levels of this enzyme in these regions (Fig. 6h; fCTX: *p = 0.0244; Fig. 6l; THAL: *p = 0.0227, 2-way ANOVA). The enzyme levels were not affected by BLG sensitization or thioperamide treatment in the hippocampus. On the other hand, neither sensitization nor thioperamide treatment resulted in significant changes in HNMT levels within any of the three brain regions analyzed for females (Fig. 6h, j, l, also Supplementary Fig. 6-1d). Although decreasing trends in HNMT levels were detected in the hippocampus and thalamus of female BLG mice with thioperamide treatment (Fig. 6, j & l), the differences from the treatment-matched sham groups did not reach statistical significance.

### Preventative Effect of Thioperamide On CMA-Associated Cortical Demyelination

**Fig. 7:**
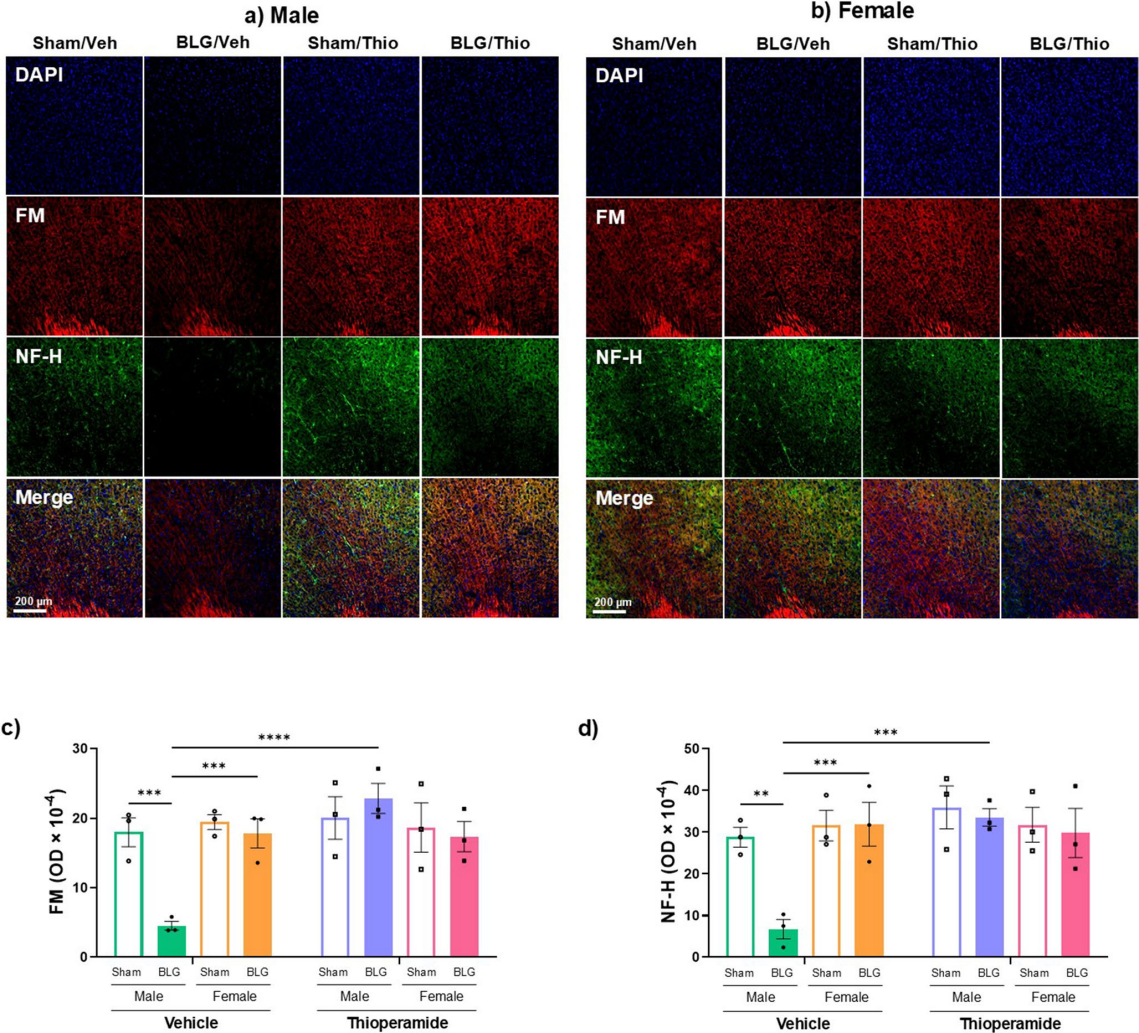
Fluorescence staining of myelin and neurofilament heavy chain in the anterior cingulate. Brain sections from male (**a**) and female (**b**) sham and BLG mice with vehicle (Veh) and thioperamide (Thio) treatment were stained with FluoroMyelin™ (FM) and neurofilament heavy chain (NF-H) for myelin sheath (red) and axonal structure (green), respectively. DAPI nuclear counterstain was used to identify cell bodies (blue). Representative photomicrographs were taken from the anterior cingulate region of the frontal cortex using a 10× objective. Scale bar = 200 μm. Staining differences among the experimental groups were compared by randomly selecting three samples from each group and quantifying the optical density (OD) of FM staining (**c**) and NF-H immunostaining (**d**) within the anterior cingulate cortex. The bars indicate the relative OD value for each treatment group ± SEM (*n* = 3). Sex-disaggregated analyses were performed by 2-way ANOVA, and asterisks between two bars indicate significance (**p < 0.01, ***p < 0.001, ****p < 0.0001)

Thioperamide treatment attenuated the CMA-associated behavioral changes more likely to be observed in BLG-sensitized male mice (Figs. 3 and 4). To assess whether the behavioral improvement reflected the protective effect of thioperamide on cortical demyelination previously reported in BLG-sensitized mice (Germundson and Nagamoto-Combs 2022), we compared the neuropathology development in sham and BLG mice without or with the H3R antagonist. We performed immunofluorescence staining of brain sections with FluoroMyelin™ (FM) to visualize myelin sheath, as well as with anti-neurofilament heavy chain (NF-H) antibody to determine the structural integrity of neuronal axons (Fig. 7, a & b). In fCTX of male BLG mice, substantially reduced immunofluorescence signals were detected for both FM and NF-H compared to sham mice. This observation was particularly salient in the anterior cingulate region but not apparent in other brain regions or large white-matter structures, including the corpus callosum. Comparisons among the sex-matched groups indicated that the immunofluorescence signals in BLG-sensitized male mice were significantly lower for both FM and NF-H than other groups (Fig. 7, c & d; FM: open vs. filled green bars, ***p = 0.0007, NF-H: open vs. filled green bars, **p = 0.0016, 2-way ANOVA), but no obvious differences were observed among the female groups. Importantly, sensitization-induced decreases in the immunofluorescence signals for both FM and NF-H in fCTX were preserved in thioperamide-treated males, indicating that CMA-associated myelin and axonal integrity of the neurons in this area were protected by the treatment (Fig. 7, c & d). These observations strongly suggested that the reduced myelination and cytoskeletal integrity were likely involved in the behavioral abnormalities that were detected in male BLG mice, and H3R inhibition effectively prevented the development of this myelin-related neuropathology.

## Discussion

We examined the potential role of H3R in CMA-associated changes by antagonizing H3R activity with thioperamide. Female mice were also included to further observe sex biases in phenotypes with BLG sensitization. Previously, we described phenotypic features of subclinically sensitized and acutely challenged male and female CMA mice by analyzing sex-disaggregated data (Smith et al. 2021). Using this method of analysis, the CMA-associated neurobehavioral changes were significant in males. Sex-aggregated comparisons also validated some sex biases in immune responses and neurobehavioral changes, suggesting that how allergen exposure affects subclinically sensitized individuals may differ between males and females.

Biological differences in sex have been known to greatly influence immune responses and thus could have impacted the pathophysiology or behavioral manifestations in our CMA mouse model. For example, females are thought to have a greater capacity to clear antigens because estrogen promotes IgG production (Kanda and Tamaki 1999). Indeed, our sex-aggregated 3-way ANOVA (Fig. 2d) and sex-disaggregated 2-way ANOVA (Supplementary Fig. 2-1e) multiple comparisons showed that female mice produced greater levels of BLG-specific IgG1 than BLG males, potentially allowing more effective allergen clearance. In contrast, males tend to produce more proinflammatory cytokines through Toll-like receptor 4 activation by pathogens (Klein & Flanagan, 2016), rendering them more susceptible to chronic, inflammation-mediated depression and cognitive decline (Allison & Ditor, 2014). It should also be noted that both mouse and human mast cells express sex hormone receptors, and estrogen and progesterone treatments trigger their degranulation and HA release in vitro (Chen et al. 2010; Jensen et al. 2010; Zaitsu et al. 2007). In addition, estrogen has been shown to regulate the central HAergic system by increasing the levels of histamine H1 receptor, and subsequently HA, in the hypothalamus (Mori et al. 2014).

**Fig. 8:**
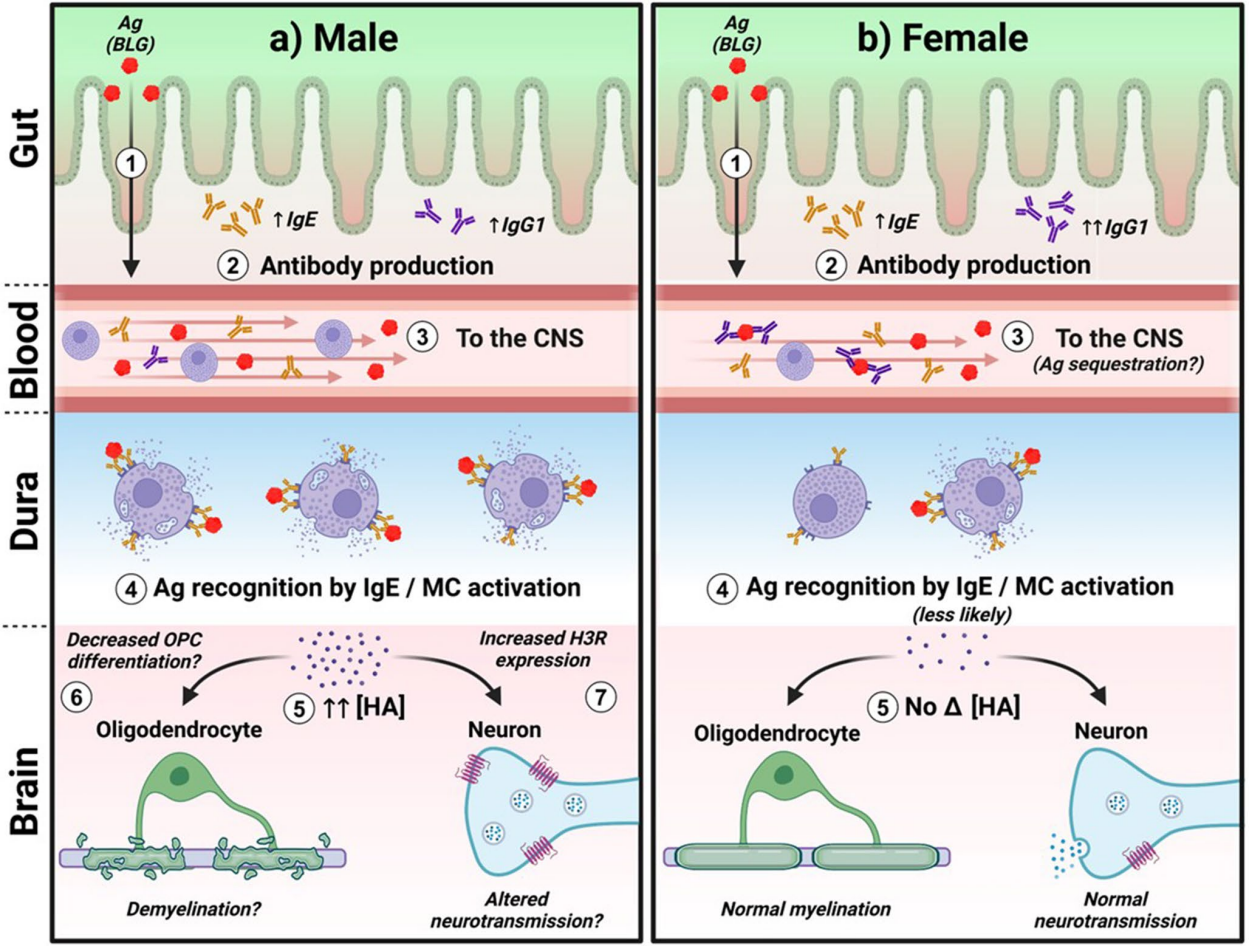
Working hypothesis with possible sex differences in CMA-associated immune responses and neuropathology. The following sequence of events is currently considered in our CMA mouse model: (1) BLG sensitization likely results in intestinal epithelial damage that allows paracellular entry of the allergen (Ag). (2) Adaptive immune responses are initiated, and allergen-specific immunoglobulins are produced. Based on our results, males (**a**) produce less allergen-specific IgG1 than females (**b**) during this process. (3) The allergen that enters the bloodstream travels to various tissues, including the meningeal and brain tissues of the CNS. In females, however, the greater amount of antigen-specific IgG1 may sequester the allergen for clearance. (4) The allergen (Ag) that reaches the dura (and other tissues) binds to the IgE coupled to mast cells (and facilitates their degranulation. (5) Degranulation of allergen-activated mast cells releases inflammatory mediators, including HA and cytokines, elevating their meningeal and parenchymal concentrations (↑↑[HA]). Released cytokines serve as chemoattractants and may recruit more immune cells to the CNS. In females, fewer immune cells may be recruited since fewer mast cells become activated, and meningeal and parenchymal concentrations of inflammatory mast cell mediators are not substantially altered (No Δ [HA]). The greater HA levels in male mice could, in turn, (6) decrease the differentiation of oligodendrocyte precursor cells (OPCs) into mature oligodendrocytes, resulting in relative demyelination. In addition, the elevated HA may increase the expression of H3R (7), potentially altering neurotransmission

While sex hormone levels and the estrous cycle were not specifically considered in our current study, additional studies are warranted, given the potential contributions of sex hormones to differential immune responses and neuropathologies. Investigating sex as a biological variable may provide a crucial key for understanding the dichotomy we observed in our male and female CMA mice. Nonetheless, based on our findings from this study, we propose our working hypothesis that male intestinal immune cells elicit greater proinflammatory responses upon encountering the allergen and gut microbes, whereas such behavior-altering responses may be dampened in females (Fig. 8).

We also tested the involvement of H3R-mediated HA signaling in CMA-associated changes since central HAergic dysregulation had been implicated in various behavioral and neuropathological manifestations. Thioperamide was used as a highly selective and potent H3R antagonist (Ki = 4 nM) (Arrang et al. 1987) with an ability to cross the BBB (Silva et al. 1997). It has been used in early studies to elucidate the functions of the HAergic system in the brain (Arrang et al. 1987). Beneficial effects of H3R modulation in animal models of neurological disorders have also been reported using different H3R antagonists, such as DL77 and SAR110894 (Delay-Goyet et al. 2016; Eissa et al. 2018). In this study, we chose to administer thioperamide via intragastric gavage, as individual oral administration allows for precise individual dosing and high bioavailability in the brain at 30 mg/kg (Perez-Garcia et al. 1999). While oral gavage during the 2 weeks of the whey protein diet could be an additional stressor, all mice, including vehicle-treated mice, were gavaged daily. In future studies, other administration routes could be considered, particularly if testing sensitive behaviors or stress-affected molecules. Additionally, while thioperamide administration was given after sensitization and during the whey diet, further testing is warranted to determine if thioperamide is effective after the onset of observable behavioral or neuropathological symptoms, or if only prophylactic treatment significantly ameliorates these changes.

Daily thioperamide treatment during allergen exposure ameliorated CMA-associated behavioral and histological phenotypes, more evidently in male mice (Figs. 3 and 4, & 7), suggesting the role of H3R in pathology development. While thioperamide also inhibits the H4 subtype of HA receptor (H4R) (Gbahou et al. 2006), H4R involvement is unlikely since its CNS expression is low (Moya-Garcia et al. 2011). In contrast, H3R is expressed ubiquitously in the brain and functions as a Gα_i/o_-protein-coupled postsynaptic heteroreceptor to regulate the release of other neurotransmitters, including acetylcholine, norepinephrine (Schlicker et al. 1999), dopamine, GABA (Jang et al. 2001), and serotonin (Fox et al. 2005) in HAergic fiber target regions. H3R antagonism increases the release of these neurotransmitters in the frontal cortex and hippocampus (Fox et al. 2005). Using other H3R-specific antagonists, such as pitolisant, will further validate the involvement of this and other centrally expressed histamine receptor subtypes, particularly H2R, which has been shown to negatively regulate oligodendrocyte differentiation in the neonatal brain (Jiang et al. 2021).

When analyzed individually by sex, thioperamide significantly improved depression-like behavior and cognitive decline in male BLG mice by decreasing their immobile time (Fig. 4b) and enhancing their recognition memory (Fig. 4h), respectively. However, it also seemed to have influenced the activity-related parameters of the sham groups with the open field test, by increasing time immobile in females (Fig. 3f) and decreasing total distance traveled and average speed in males (Fig. 3j & l). This result may be explained by the effect of thioperamide to induce a short period of decreased mobility for at least 1.5 h after administration (Perez-Garcia et al. 1999). Because the open field test was performed 1 to 3 h after the mice were given thioperamide, the possible short-term effect of the antagonist could have produced a relative decrease in overall activity.

Although it is unlikely that thioperamide acted on centrally expressed H4R, it could have elicited its behavior-modifying effect through H4R on peripheral immune cells. H4R inhibition by thioperamide is known to modulate the activities of immune cells expressing this receptor subtype (Dib et al. 2014), raising the possibility that thioperamide influenced peripheral immune cells involved in antibody production and/or HA release. Such potential peripheral effects of the H3R antagonist limit interpretations in our current study; the use of H4R-specific ligands, non-BBB-permeable H3R antagonists, or intracerebroventricular administration to minimize any impact on peripheral immune cells should clarify this point.

Our current hypothesis is that HA released into the intracranial space from activated dural mast cells dysregulates the central HAergic system by superfluously elevating HA concentration in the brain (Germundson and Nagamoto-Combs 2022). The sensitization-associated increases in degranulation of IgE-coupled dural mast cells (Fig. 5n) and HA + NMHA levels in the frontal cortex and thalamus (Fig. 6, b & f) further support this hypothesis. Because dietary allergens can be detected in the sera and brain tissues of BLG-sensitized mice (Germundson and Nagamoto-Combs 2022), we propose a dural mast cell degranulation mechanism as follows: (1) sensitization-induced IgE becomes associated with the FcεRI on dural mast cells; (2) IgE-coupled mast cells encounter the allergen in the circulation; and (3) mast cell degranulation is triggered via the allergen-IgE-FcεRI signaling. In females, circulating allergen may be more likely sequestered by allergen-specific IgG1, which is produced at a greater level than in males (Fig. 2d). The lower number of total IgE^+^ mast cells and the lower percentage of degranulated IgE^+^ mast cells in females corroborate this notion (Supplementary Fig. 5-2, b & f).

To examine whether central HA is also regulated through metabolism, we quantified HNMT, the only HA metabolic enzyme expressed in the brain to control HA availability (Yoshikawa et al. 2019). Contrary to our expectation that HA turnover would be higher in female mice to explain their lower HA levels measured by LC-MS (Supplementary Fig. 6-2), males and females expressed roughly equivalent HNMT levels (Fig. 6, g-l), except that the enzyme levels in the thalamus of BLG-sensitized males were significantly greater than their female counterpart (Fig. 6, k & l, Supplementary Fig. 6-1c). Interestingly, thioperamide effectively prevented the HNMT induction in male fCTX and THAL, suggesting a regulatory role of H3R signaling in HNMT expression.

The increases in the amounts of HA + NMHA in some brain regions of male BLG mice detected by ELISA and the decreases in HA alone measured by LC-MS in these animals led us to deduce that the elevated signals from the HA + NMHA ELISA in this group were largely from NMHA (see Fig. 6 and Supplementary Fig. 6-2). However, because we used two detection methodologies with different sensitivity and specificity that required method-specific sample processing, the direct comparison of the analyte levels determined by ELISA and LC-MS poses a limitation in this interpretation. The use of dissected brain regions for ELISA or the entire right brain hemisphere also adds a confounding factor. Individual measurements of HA and NMHA by LC-MS in targeted brain regions would provide more precise information regarding HA metabolism. Thus, further investigations are warranted to fully understand the dynamics of the central HAergic system and how it is differentially regulated in male and female brains.

The most striking effect of thioperamide in this study was the improvement of CMA-associated demyelination in male BLG mice (Fig. 7, red “FM”). Previously, we used Black Gold II myelin stain and reported significant cortical demyelination in the motor and somatosensory regions of the cerebral cortex in male BLG mice after 2 weeks of whey-protein consumption (Germundson and Nagamoto-Combs 2022). Cortical demyelination was again detected with FluoroMyelin in this study, highlighting the anterior cingulate cortex as the most notable region of demyelination (Fig. 7a). The sensitivity or resolution of the two myelin dyes might have resulted in the differences in the highlighted cortical regions. Nonetheless, the result confirmed the consistency of this neuropathology in our CMA model. We also showed decreased NF-H immunoreactivity in male BLG mice (Fig. 7, green). Because NF-H is a cytosolic structural protein that can be regulated in varying physiological and pathological conditions (Shaw et al. 2005), the loss of NF-H signals in BLG-sensitized mice likely reflects downregulation and/or degradation of this protein in the axons, potentially triggered by demyelination (Lovas et al. 2000; Schirmer et al. 2011) rather than axonal loss. No apparent change in myelin density or NF-H immunoreactivity was observed in female BLG mice (Fig. 7, b-d), underscoring the sex-biased phenotype manifestations of CMA-associated neuropathology in our mouse model.

Importantly, the CMA-associated cortical demyelination in male BLG mice was markedly diminished with H3R inhibition with thioperamide (Fig. 7, a-c). Myelin renewal is a physiological process performed by mature oligodendrocytes to maintain axonal conductance (Williamson and Lyons 2018). Thus, it is possible that the HA/H3R system regulates this process by stimulating the active breakdown of myelin and/or inhibiting remyelination via slowing of oligodendrocyte maturation. Indeed, other studies using models of material inflammation-induced white matter injury (Rangon et al. 2018) and cuprizone-induced demyelination (Chen et al. 2017) have also reported increases in myelin basic protein and proteolipid proteins after treatment with H3R antagonists other than thioperamide. Although more direct evidence supporting the causative role of HA in demyelination is required, elevated HA has been found in the cerebrospinal fluid (CSF) of multiple sclerosis patients (Tuomisto et al. 1983), and individuals with overactive mast cell activity can present with white matter damage (Haenisch and Molderings 2018). Furthermore, in vitro and in vivo studies have demonstrated that H3R is expressed by oligodendrocytes, and its activation inhibits oligodendrocyte precursor differentiation (Chen et al. 2017). Thus, it is feasible to postulate that thioperamide may have prevented HA/H3R-mediated slowing of oligodendrocyte maturation in BLG-sensitized CMA mice.

Additional studies, including CSF analyses, are required to further elucidate the demyelination process in our CMA model. It is also important to directly compare our findings to human multiple sclerosis and other demyelinating conditions as we investigate allergen exposure as a potential extrinsic factor that triggers chronic neuroinflammation in intrinsically predisposed individuals. Human conditions to be compared could also include mast cell activation syndromes, in which afflicted patients show inappropriate mast cell activation with higher rates of neuropsychiatric conditions than the general public (Sagües-Sesé et al. 2023). One study reported that approximately one-half of their mastocytosis patient cohort presented with punctuated white matter abnormalities on magnetic resonance imaging (Haenisch and Molderings 2018). Provided that our CMA model with C57BL/6J mice on a whey-protein diet closely simulates repeated consumption of allergens by subclinically sensitized or allergen-tolerant individuals as we previously proposed (Brishti et al. 2022; Germundson and Nagamoto-Combs 2022; Germundson et al. 2018; Smith et al. 2019, 2021), allergen avoidance would likely prevent neurobehavioral pathology development. Furthermore, an H3R antagonist may decrease the possibility of neurodegenerative events in sensitized but tolerant individuals who do not strictly avoid allergen-containing foods.

## Supplementary Information

Below is the link to the electronic supplementary material.

Supplementary Material 2-1 (JPG. 261 KB) 

Supplementary Material 2-2 (JPG. 161 KB)

Supplementary Material 2-3 (JPG. 204 KB)

Supplementary Material 3-1 (JPG. 208 KB)

Supplementary Material 3-2 (JPG. 128 KB)

Supplementary Material 3-3 (JPG. 285 KB)

Supplementary Material 4-1 (JPG. 143 KB )

Supplementary Material 4-2 (JPG. 305 KB)

Supplementary Material 4-3 (JPG. 130 KB)

Supplementary Material 5-1 (JPG. 149 KB)

Supplementary Material 5-2 (JPG. 337 KB)

Supplementary Material 5-3 (JPG. 198 KB)

Supplementary Material 6-1 (JPG. 284 KB)

Supplementary Material 6-2 (JPG. 177 KB)

## Data Availability

Data presented in this study is available on request from the corresponding author.
