# Supplementary figures and images for: Histamine H3 Receptor Antagonist, Thioperamide, Improves Behavioral and Neuropathological Changes Associated with Subclinical Hypersensitivity to a Cow’s Milk Allergen
Source: J Neuroimmune Pharmacol. 2025 Dec 19;20(1):110. doi: 10.1007/s11481-025-10256-9 (PMC12717242; doi:10.1007/s11481-025-10256-9)

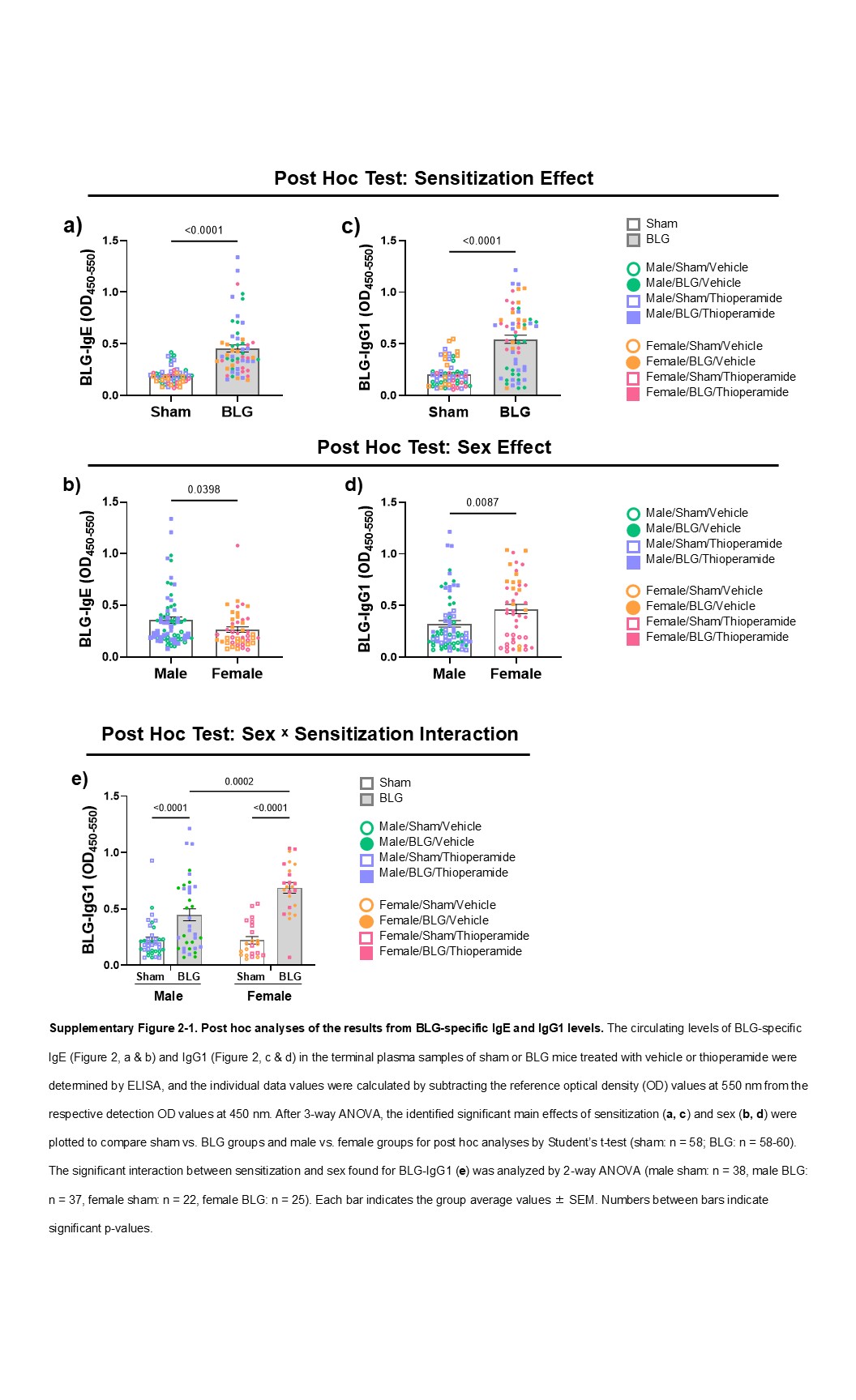

Supplement: Supplementary file 1 — Supplementary Material 2-1 (JPG. 261 KB) [file 11481_2025_10256_MOESM1_ESM.jpg]

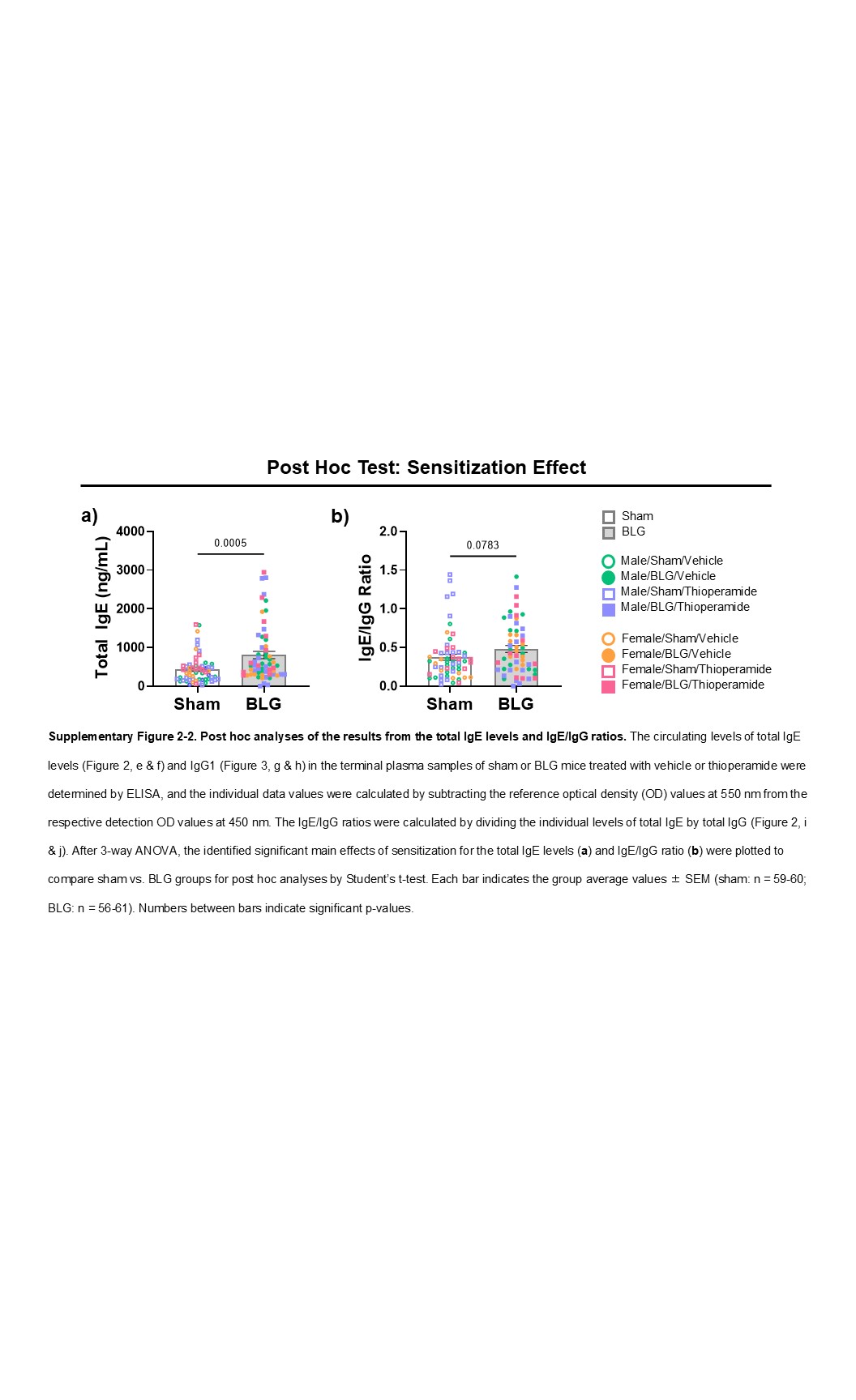

Supplement: Supplementary file 2 — Supplementary Material 2-2 (JPG. 161 KB) [file 11481_2025_10256_MOESM2_ESM.jpg]

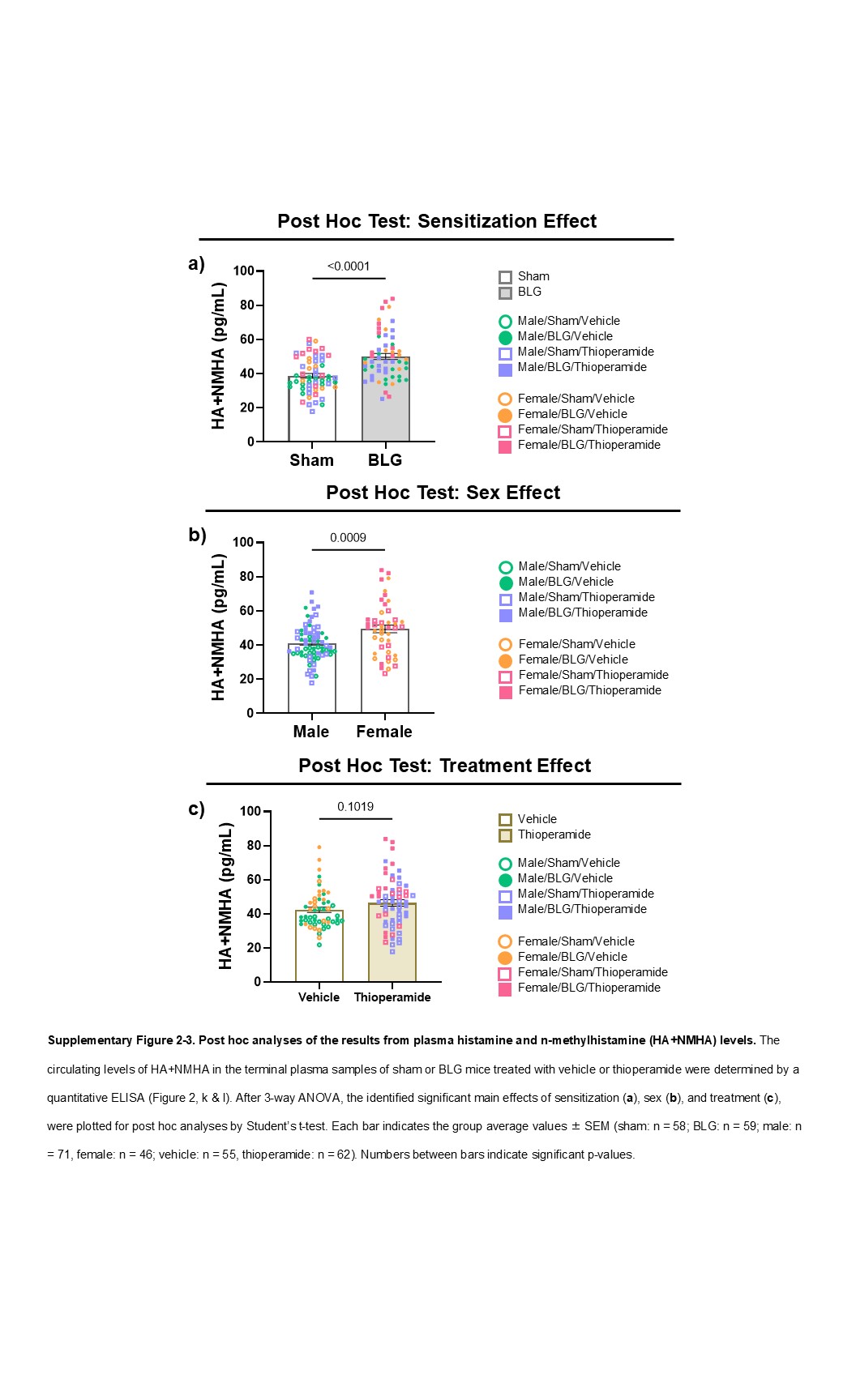

Supplement: Supplementary file 3 — Supplementary Material 2-3 (JPG. 204 KB) [file 11481_2025_10256_MOESM3_ESM.jpg]

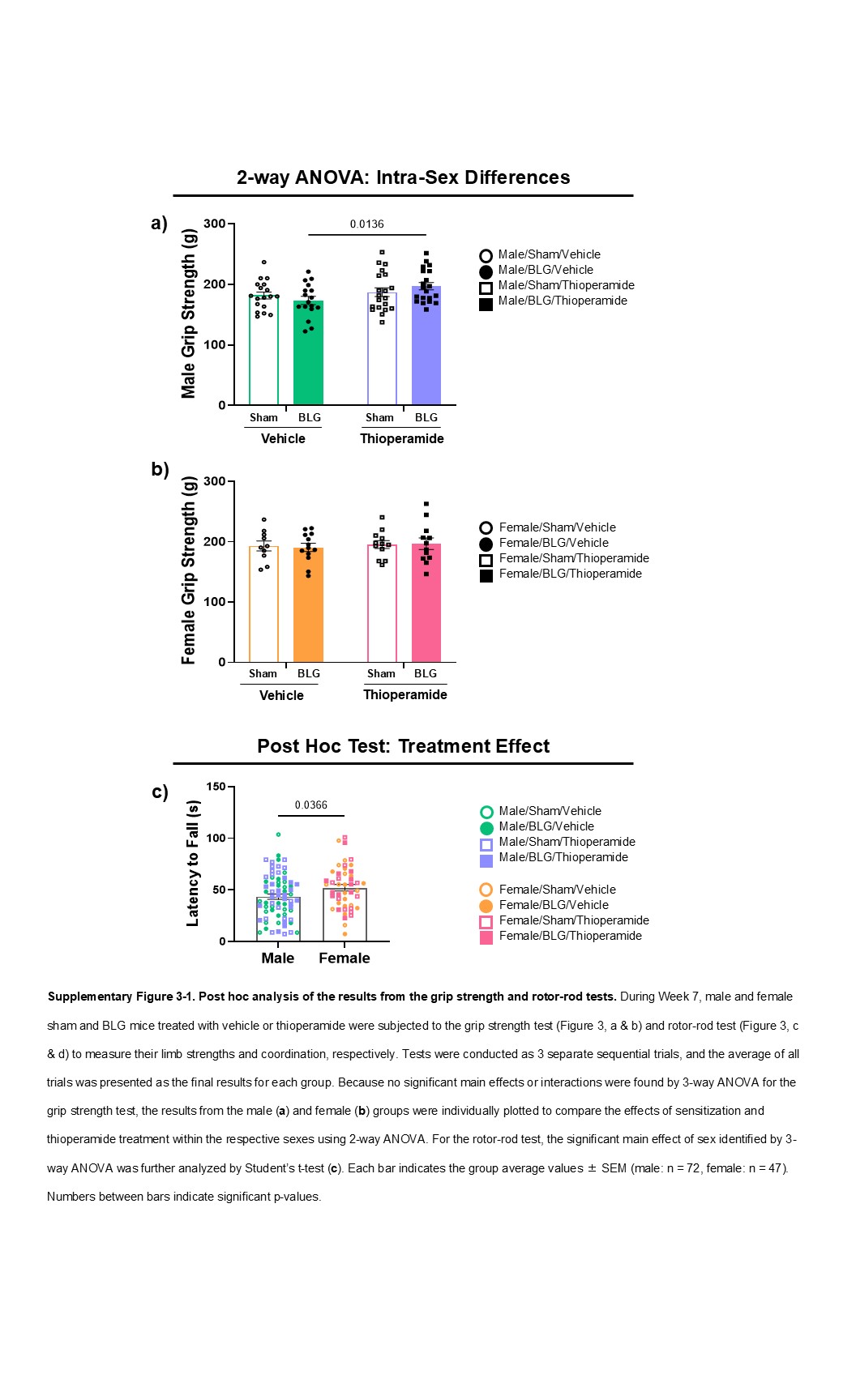

Supplement: Supplementary file 4 — Supplementary Material 3-1 (JPG. 208 KB) [file 11481_2025_10256_MOESM4_ESM.jpg]

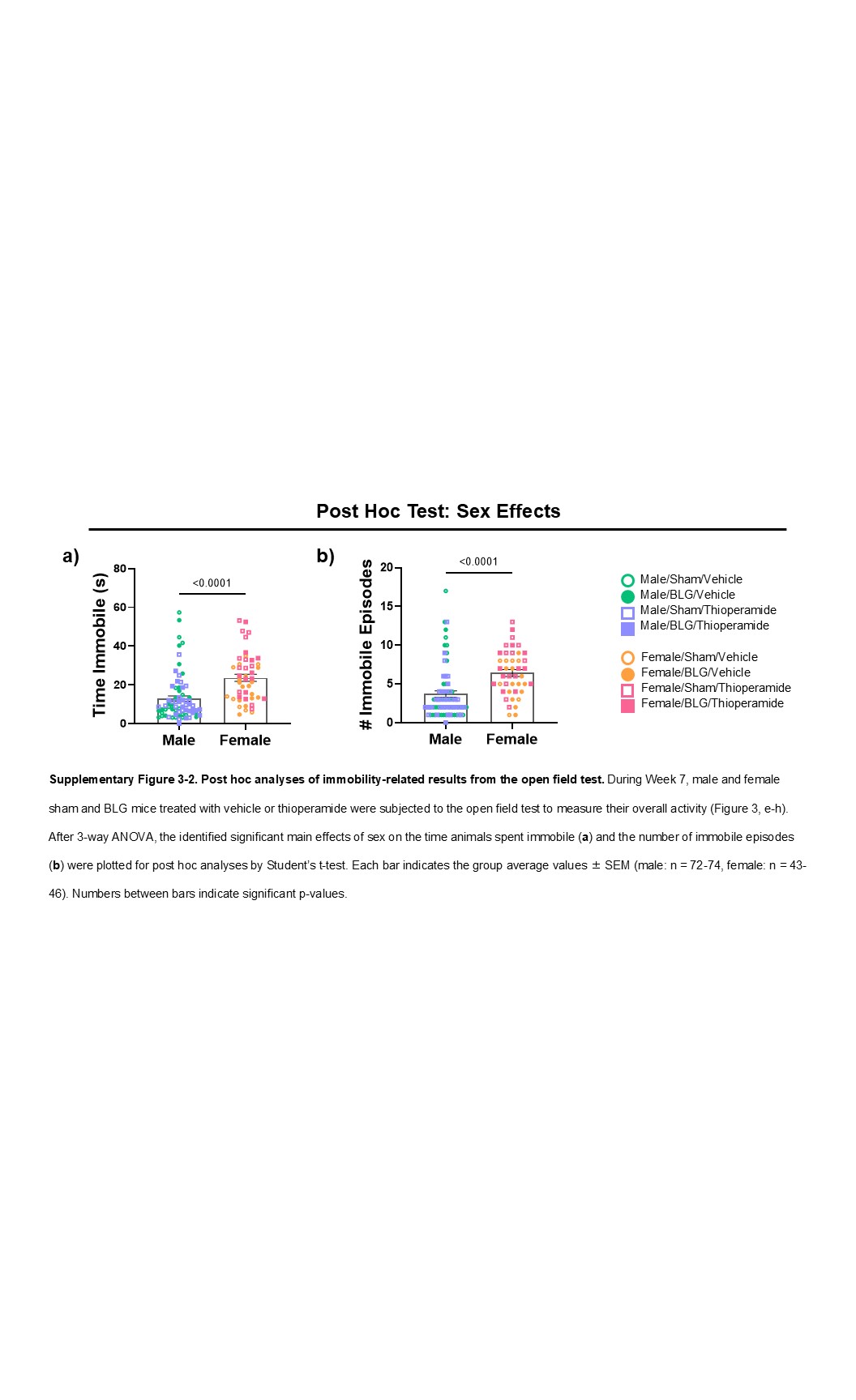

Supplement: Supplementary file 5 — Supplementary Material 3-2 (JPG. 128 KB) [file 11481_2025_10256_MOESM5_ESM.jpg]

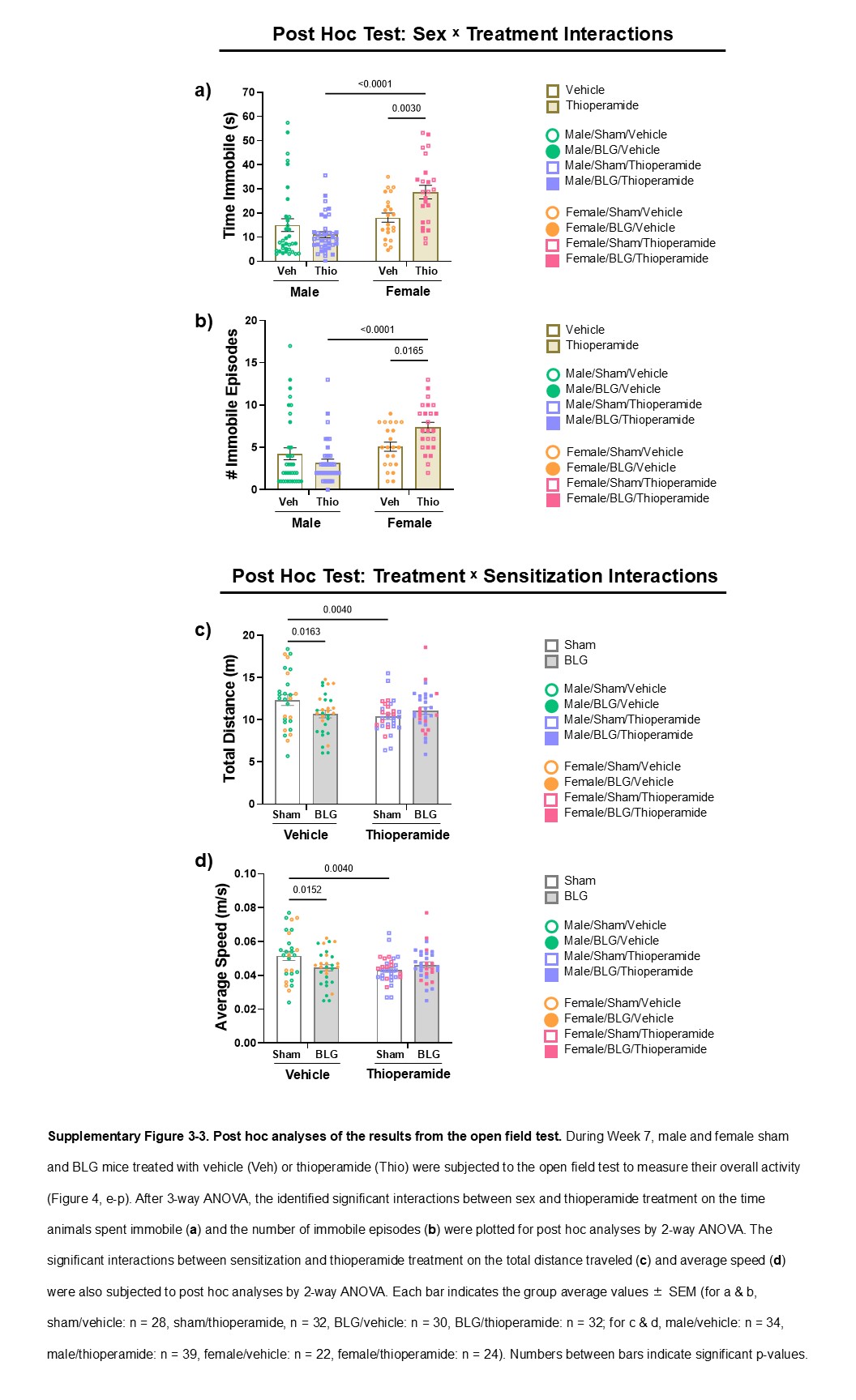

Supplement: Supplementary file 6 — Supplementary Material 3-3 (JPG. 285 KB) [file 11481_2025_10256_MOESM6_ESM.jpg]

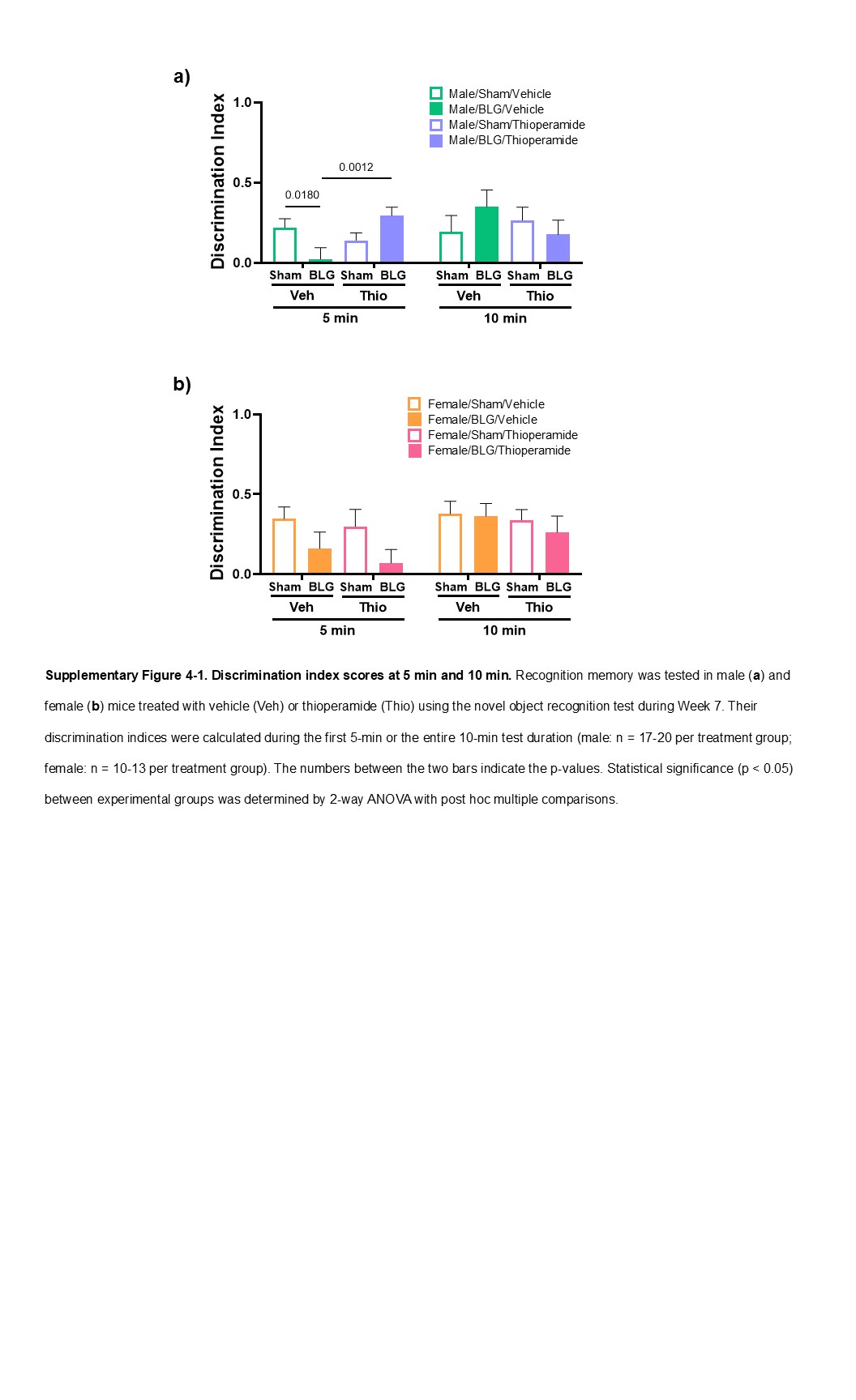

Supplement: Supplementary file 7 — Supplementary Material 4-1 (JPG. 143 KB ) [file 11481_2025_10256_MOESM7_ESM.jpg]

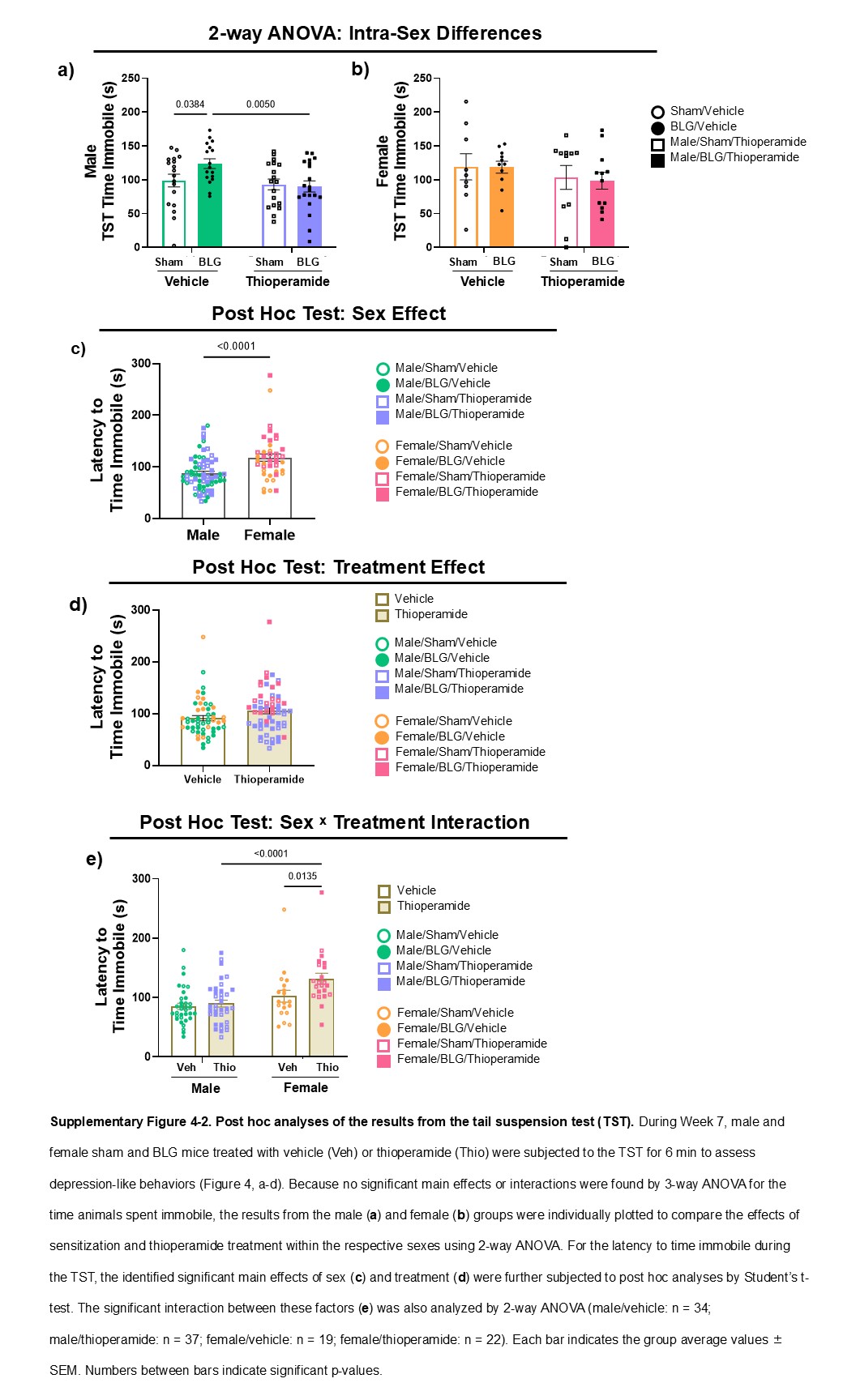

Supplement: Supplementary file 8 — Supplementary Material 4-2 (JPG. 305 KB) [file 11481_2025_10256_MOESM8_ESM.jpg]

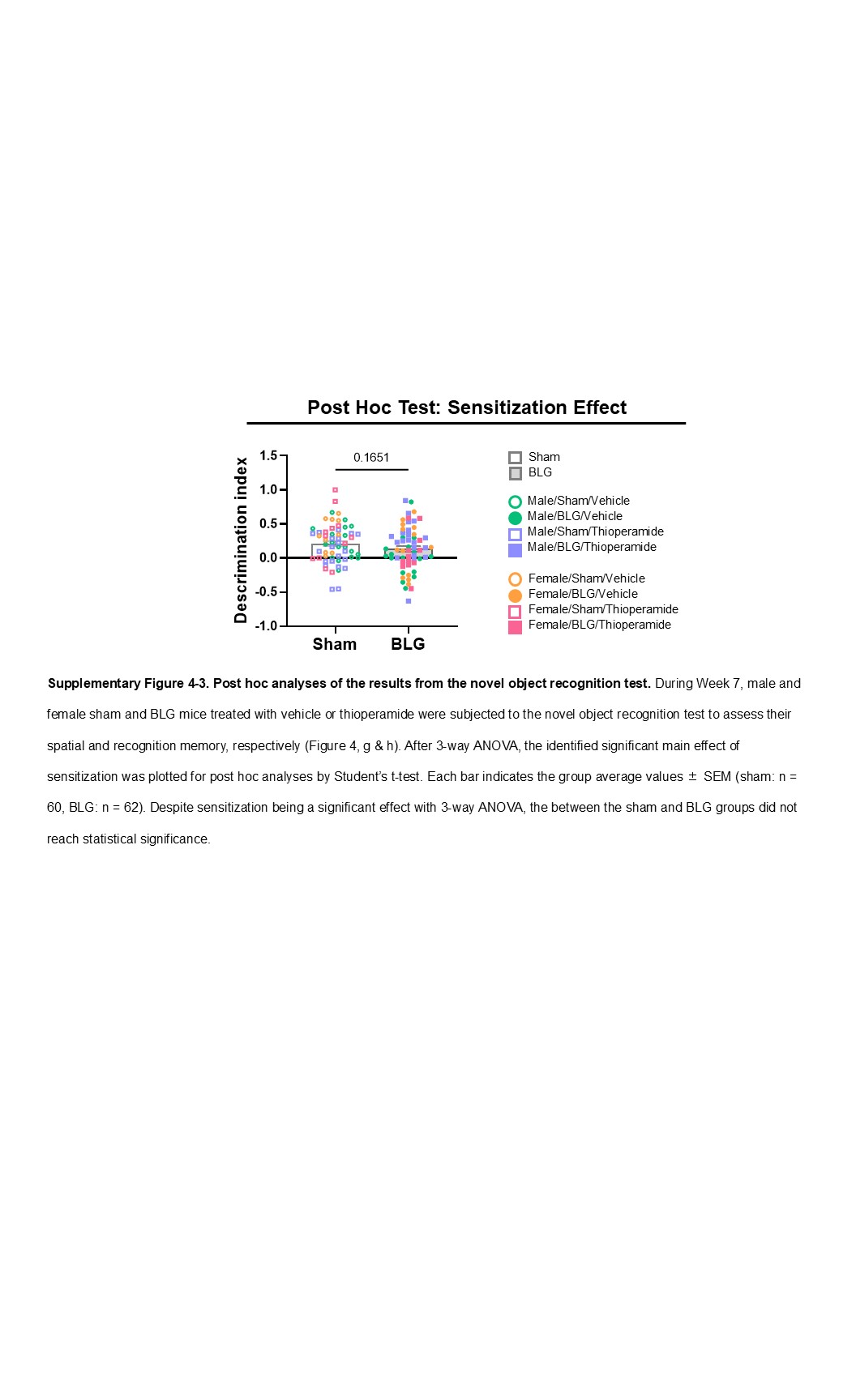

Supplement: Supplementary file 9 — Supplementary Material 4-3 (JPG. 130 KB) [file 11481_2025_10256_MOESM9_ESM.jpg]

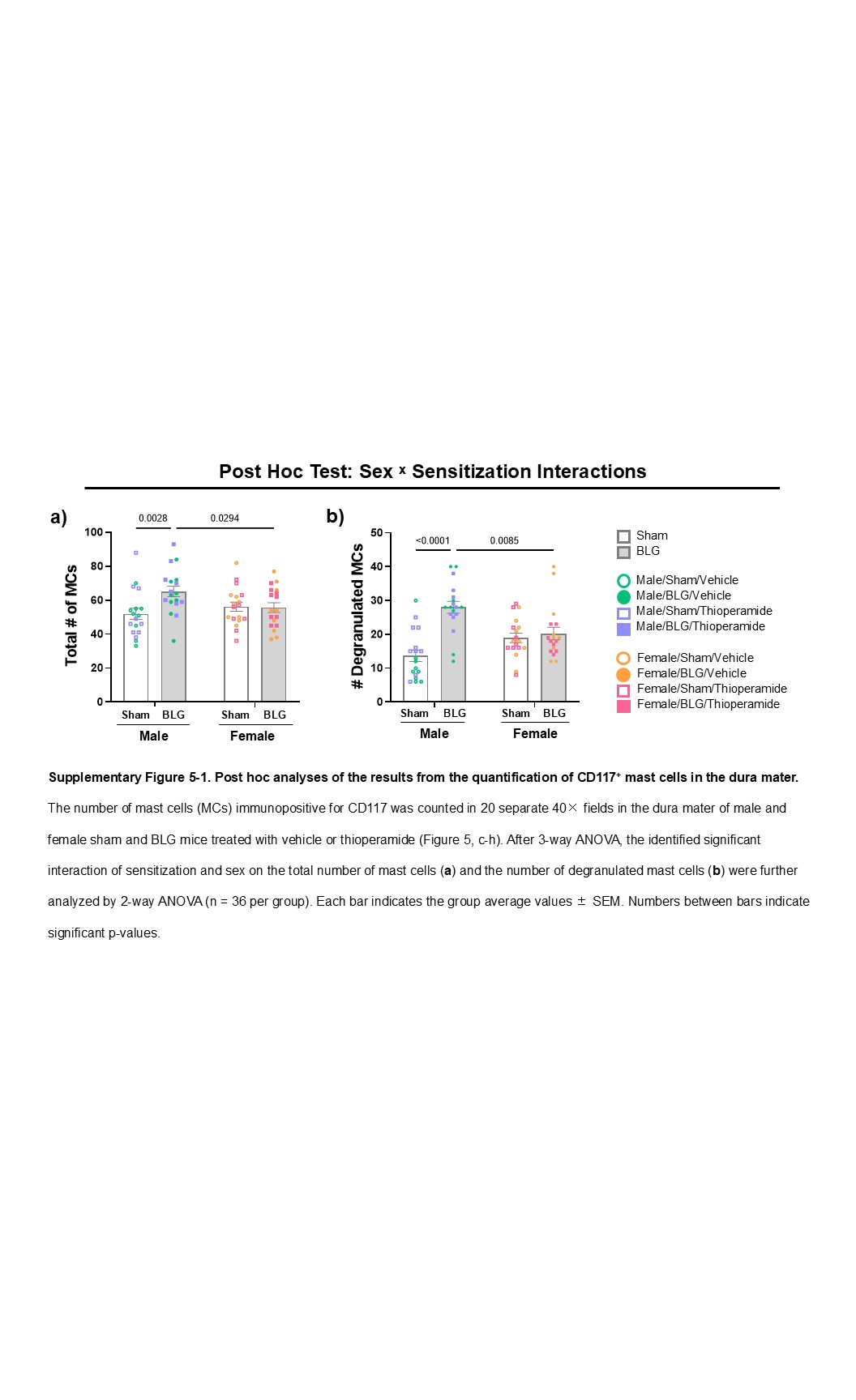

Supplement: Supplementary file 10 — Supplementary Material 5-1 (JPG. 149 KB) [file 11481_2025_10256_MOESM10_ESM.jpg]

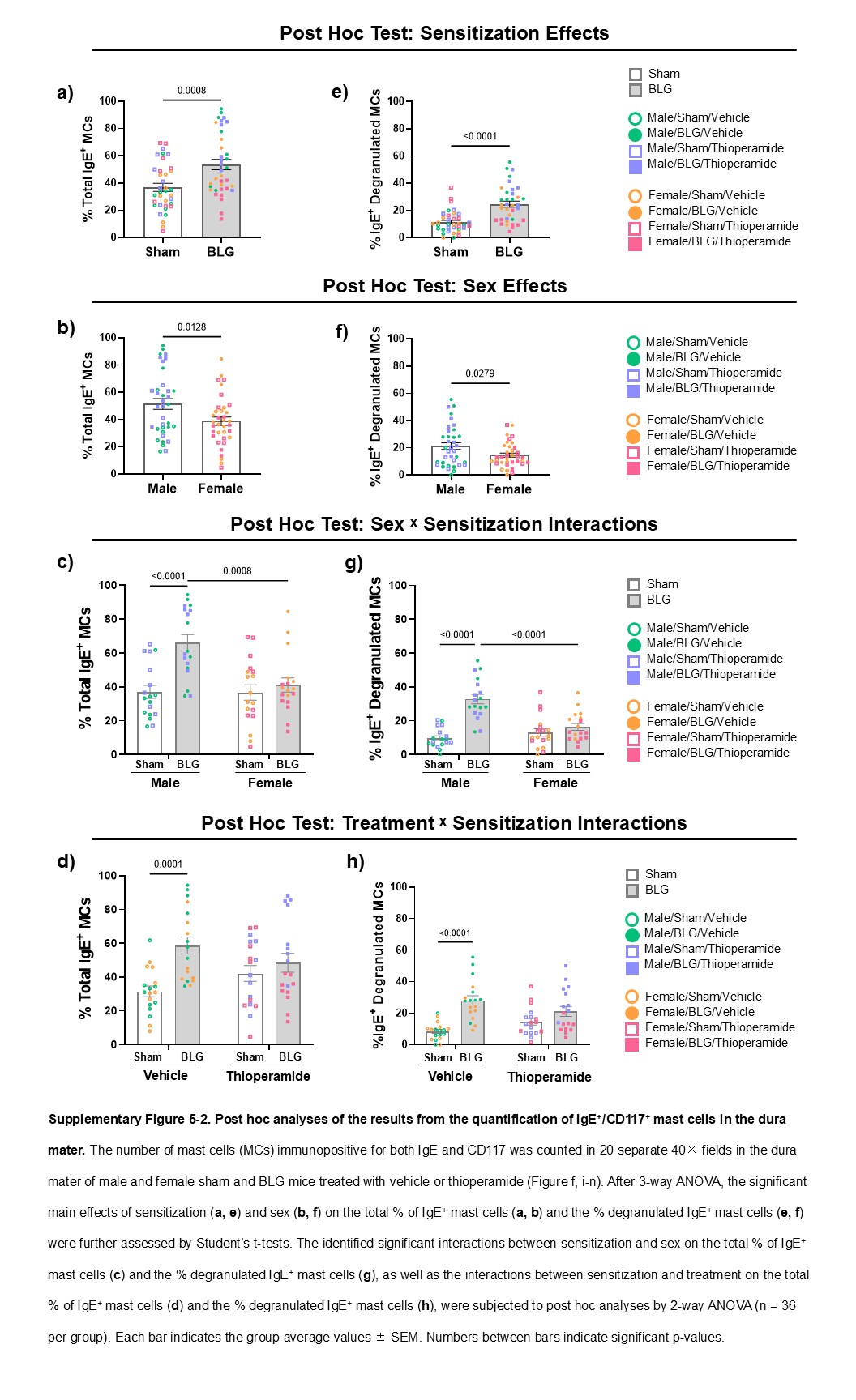

Supplement: Supplementary file 11 — Supplementary Material 5-2 (JPG. 337 KB) [file 11481_2025_10256_MOESM11_ESM.jpg]

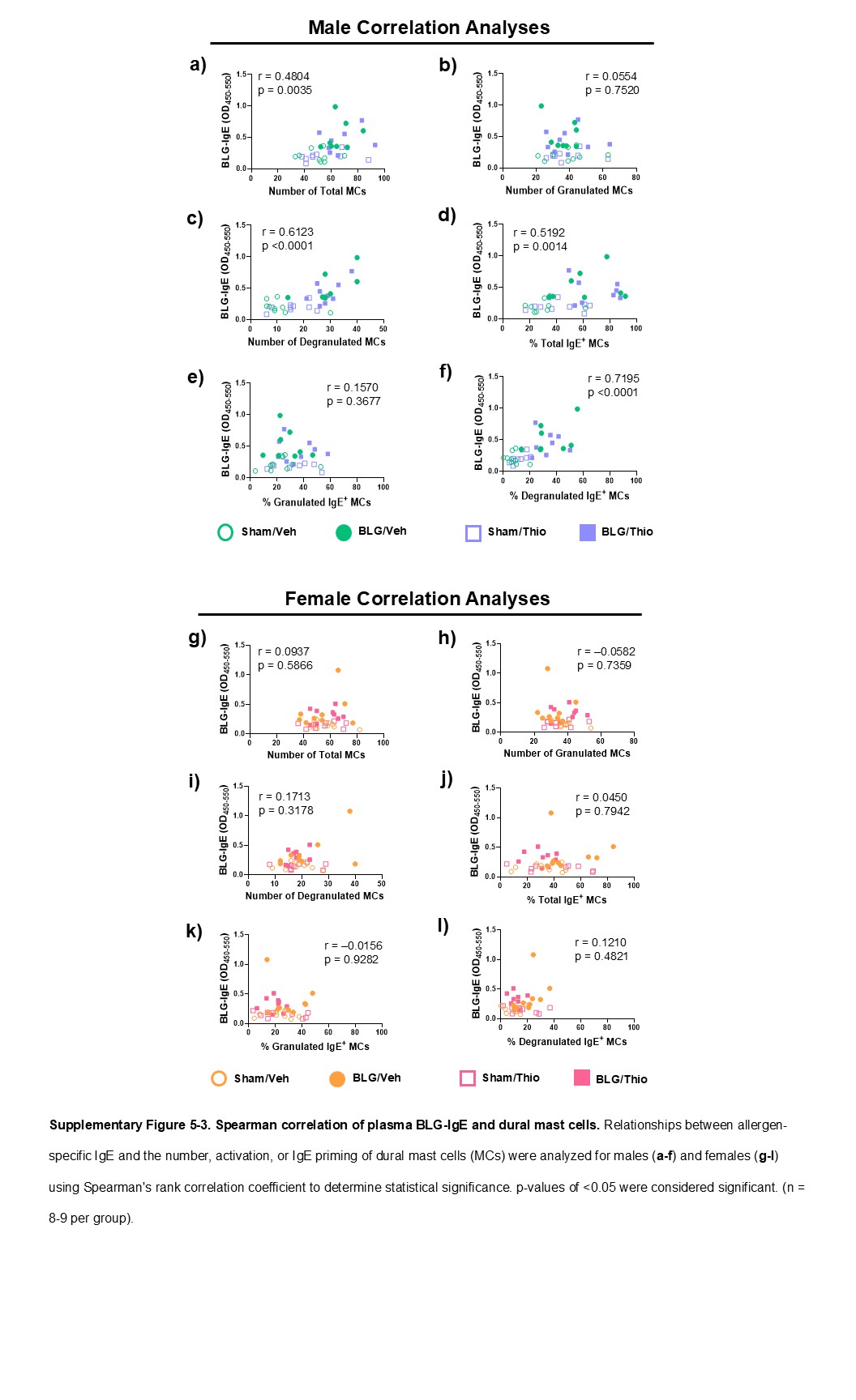

Supplement: Supplementary file 12 — Supplementary Material 5-3 (JPG. 198 KB) [file 11481_2025_10256_MOESM12_ESM.jpg]

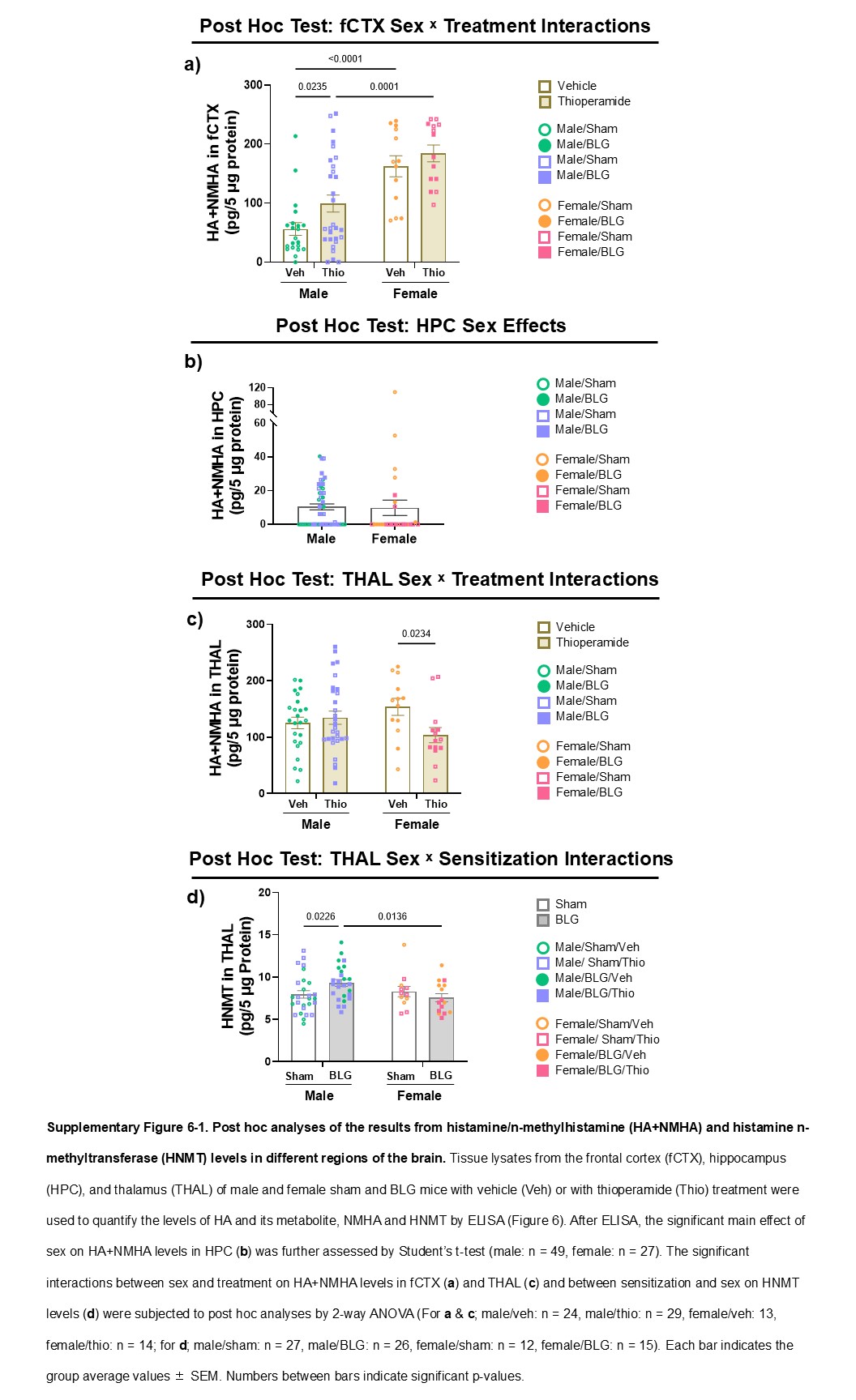

Supplement: Supplementary file 13 — Supplementary Material 6-1 (JPG. 284 KB) [file 11481_2025_10256_MOESM13_ESM.jpg]

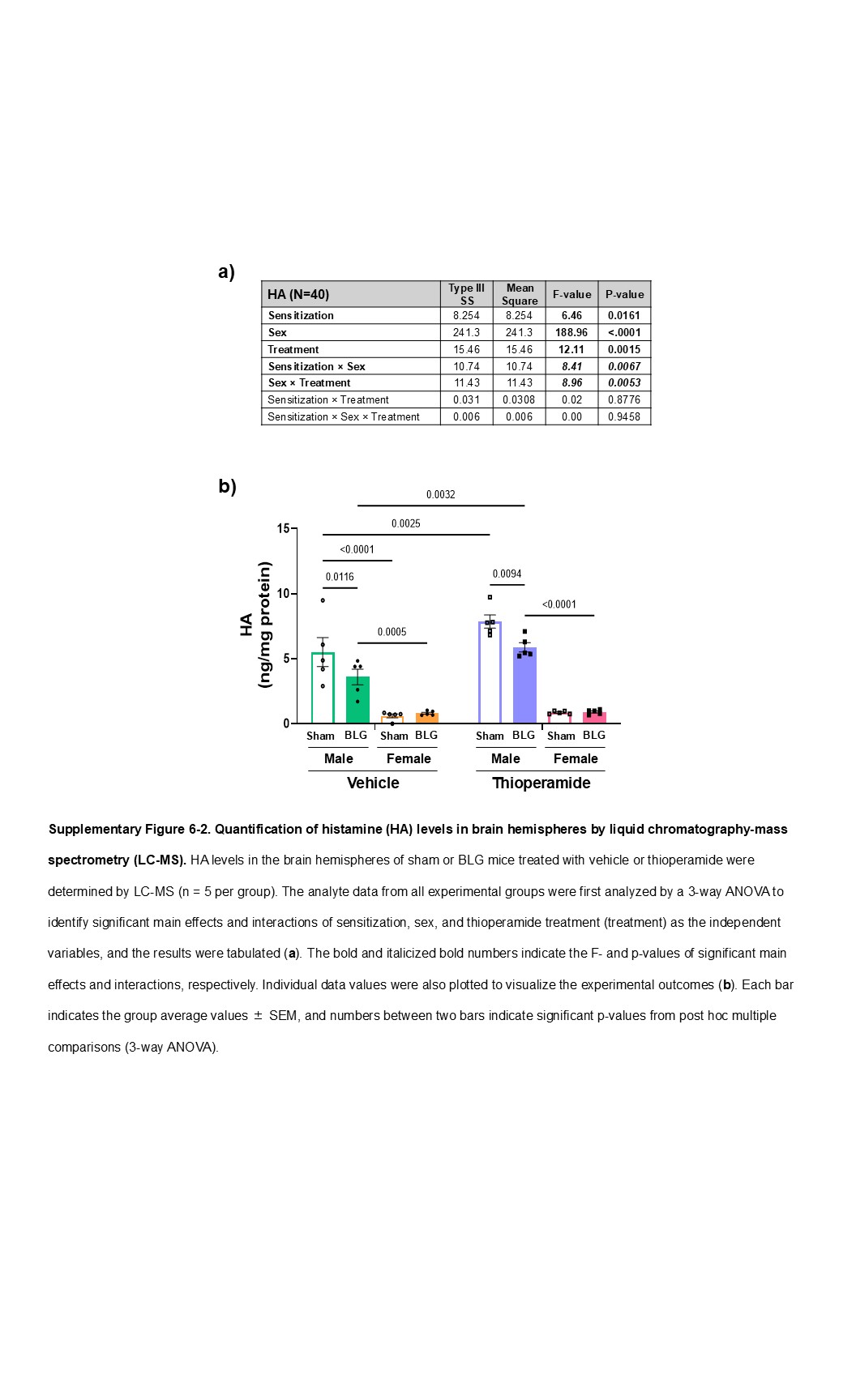

Supplement: Supplementary file 14 — Supplementary Material 6-2 (JPG. 177 KB) [file 11481_2025_10256_MOESM14_ESM.jpg]
